# Applications of Functionalized Carbon Nanotubes for the Therapy and Diagnosis of Cancer

**DOI:** 10.3390/polym9010013

**Published:** 2017-01-04

**Authors:** Yongsung Hwang, Sung-Hoon Park, Jin Woo Lee

**Affiliations:** 1 Soonchunhyang Institute of Medi-bio Science, Soonchunhyang University, Cheonan-si, Chungcheongnam-do 31151, Korea; yshwang0428@sch.ac.kr; 2 Institute of Tissue Regeneration, College of Medicine, Soonchunhyang University, Cheonan-si, Chungcheongnam-do 31151, Korea; 3 Department of Mechanical Engineering, Soongsil University, Dongjak-gu, Seoul 06978, Korea; 4 Department of Molecular Medicine, School of Medicine, Gachon University, Incheon 21999, Korea

**Keywords:** carbon nanotubes (CNTs), cancer, therapy, nano-carriers, functionalization

## Abstract

Carbon nanotubes (CNTs) are attractive nanostructures that serve as multifunctional transporters in biomedical applications, especially in the field of cancer therapy and diagnosis. Owing to their easily tunable nature and remarkable properties, numerous functionalizations and treatments of CNTs have been attempted for their utilization as hybrid nano-carriers in the delivery of various anticancer drugs, genes, proteins, and immunotherapeutic molecules. In this review, we discuss the current advances in the applications of CNT-based novel delivery systems with an emphasis on the various functionalizations of CNTs. We also highlight recent findings that demonstrate their important roles in cancer imaging applications, demonstrating their potential as unique agents with high-level ultrasonic emission, strong Raman scattering resonance, and magnetic properties.

## 1. Introduction

Cancer is defined as the uncontrolled growth of cells that infiltrate and ultimately destroy normal tissues [1]. Several mutagenesis steps and the uncontrollability of cell growth and division allow cancer cells to acquire the properties of self-sufficiency in growth signals, unlimited proliferation potential, and resistance to signals from normal cells that would otherwise halt their proliferation or induce apoptosis [2]. Tumors can also evolve to acquire additional support by interactions among surrounding stromal cells, including those that promote angiogenesis, thus helping them evade the immune system, resulting in metastasis [3]. Every year, an estimated 14 million new cases of cancer are diagnosed, and 8.9 million people die of the disease [1]. Lung, stomach, liver, colon, and breast cancers are the most frequent causes of cancer-related death. Although cancer research technologies have advanced enormously, methods to detect cancer at an early stage and cancer-specific therapy that does not damage healthy cells are urgently needed.

Aono and Ariga [4,5,6] recently introduced an emerging new concept for nanotechnology, termed nanoarchitectonics. This novel concept has introduced a paradigm shift for material fabrication, which is an innovative technology to integrate various factors and effects, and merge them into functional nanomaterials and nanosystems. This technology covers a wide range of applications in the fields of electronics, mechanics, materials, cosmetics, packaging, textiles, optical devices, and solar cells. In recent years, this technology has been expanded toward applications in biomedical fields, including cultivation, detection, diagnosis, imaging, and various cancer therapeutics [7,8].

Nanoparticles (NPs) are the most common nanotechnology-based products used in biomedicine, which can be either organic or inorganic [9,10]. Organic NPs include liposomes, dendrimers, chitosan, viruses, solid-lipid NPs, and polymeric NPs; they have long been studied for their potential in cancer diagnosis and therapy. Inorganic NPs include gold, silver, silica, magnetic particles, ceramic particles, quantum dots (QDs), and carbon particles. Inorganic NPs have also been identified as promising vector elements in cancer treatment.

**Figure 1:**
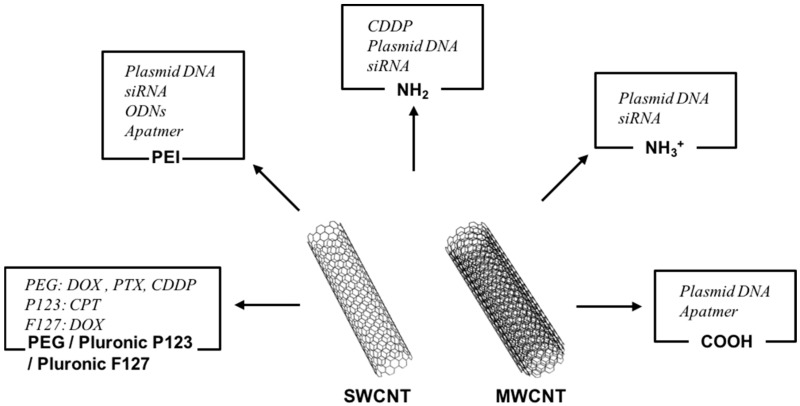
Schematics of a single-walled carbon nanotube (SWCNT) and multi-walled carbon nanotube (MWCNT) (center), and various functionalizations/applications of CNTs for cancer therapy.

Carbon nanotubes (CNTs) are hollow fibrous carbon molecules that were first observed in the soot of arc-burned graphite rods [11] (Figure 1). Owing to their high aspect ratio, large surface area, rich surface chemical functionality, and size stability at the nano-scale, CNTs have been extensively investigated for various applications such as electromagnetic interference shielding, storage capacitors, membranes, and structural reinforcement [12]. In particular, CNTs are considered a practical choice of material in biomedicine applications, and are attractive candidate transporters for the delivery of biomolecules and drugs [13]. Indeed, with chemical functionalization, CNTs have been used as nano-carriers to transport anticancer drugs, genes, and proteins for chemotherapy. They have also been used to transport agents for near-infrared (NIR) fluorescence emission, photoacoustic (PA) imaging, Raman scattering, and magnetic resonance imaging (MRI). Therefore, in this review, we highlight representative applications of CNTs for cancer therapy and diagnosis, focusing on their functions as delivery vectors and contrast enhancers in biomedical imaging modalities. The different approaches and corresponding comparisons between CNT-based and polymeric NP-based applications described in this review are summarized in Table 1 and Table 2.

## 2. Carbon Nanotubes (CNTs) as Carriers of Molecules, Genes, and Proteins

Since CNTs have high aspect ratios, very small sizes, and high surface areas, they can adsorb and/or conjugate to various therapeutic molecules. The needle-like shape of CNTs and their ease of tunable functionalization are well known to facilitate their internalization into target cells. Therefore, CNTs have been identified as promising nano-carriers for the delivery of drugs, genes, and proteins. Specifically, the intrinsic nature of the safety of vesicle-based carriers such as liposomes has greatly promoted the utilization of CNTs in cancer more than other diseases, and thus the majority of the research concerning CNT-based nano-carriers has focused on the delivery of anticancer agents [13].

### 2.1. CNTs as Carriers of Anticancer Molecules

Although chemotherapy is generally coupled with other treatment techniques such as radiation and surgery to reduce the number and size of tumors, it could cause undesirable toxicity given that cancer drugs tend to have a narrow therapeutic window, show non-specificity to cancer cells, and require increased dosages due to the development of drug resistance by cancer cells [64]. Therefore, new methods to deliver anticancer molecules specifically to tumors, reduce side effects, and improve therapeutic efficacy are in high demand. In this section, we emphasize current approaches in applications of CNT-based materials as novel agents to deliver anticancer drugs.

**Figure 2:**
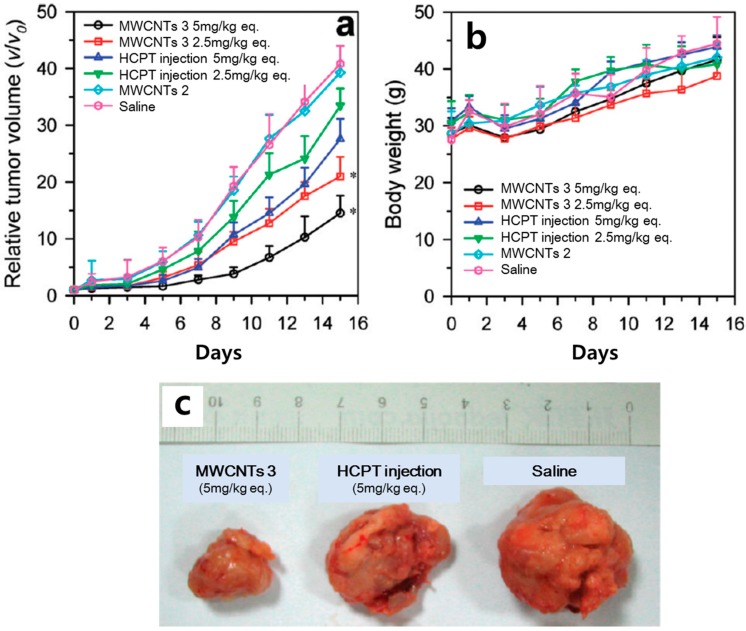
(**a**) In vivo antitumor effect obtained from treatment with multi-walled carbon nanotubes (MWCNTs), 10-hydroxycamptothecin (HCPT), and saline (control) in a mouse model, expressed as the average values of the relative tumor volume *v*/*v*_0_ (where *v* denotes the tumor volume at test time points and *v*_0_ denotes the corresponding initial tumor volume at the beginning of treatment). * *p* < 0.05 (versus the HCPT injection group at the equivalent dose from the 5th day). (**b**) Change in body weight of each group during the experiments. Data in (**a**,**b**) are presented as mean ± SD (*n* = 8); (**c**) Typical photographs of excised sarcomas from mice on the 16th day after treatments with MWCNTs 3 (5 mg/kg equiv.), HCPT injection (5 mg/kg equiv.), and saline. Reprinted with permission from Ref. [14]. Copyright (2016) American Chemical Society.

Wu et al. [14] developed a drug delivery system based on multi-walled CNTs (MWCNTs) by combining them with the antitumor agent 10-hydroxycamptothecin (HCPT). They used hydrophilic diaminotriethylene glycol as the spacer between MWCNTs and HCPT. Their HCPT-MWCNT conjugates showed remarkably improved antitumor activity compared with that of clinical HCPT formulations, both in vitro and in vivo (Figure 2). Using in vivo single-photon emission-computed tomography techniques and ex vivo gamma-scintillation counting analyses, they discovered that these conjugates were able to circulate for longer periods of time in the blood and were accumulated specifically in the tumor area. In cytotoxicity tests using human gastric carcinoma MKN-28 cells, the HCPT–CNT conjugate achieved a higher killing rate of cancer cells than obtained with injection of lyophilized HCPT at the same dose.

There have been numerous attempts to efficiently carry water-insoluble anticancer drugs by incorporating the hydrophilic moieties into CNTs. For example, Sahoo et al. [15] used a hydrophilic material, poly (vinyl alcohol) (PVA), to functionalize MWCNTs and graphene oxide (GO) to load and deliver an anticancer drug, camptothecin (CPT). They loaded CPT into PVA-MWCNTs and PVA-GO via π–π interactions, and investigated the cytotoxicity of the conjugates using human breast cancer cells (MDA-MB-231) and a skin tumor cell line (A-5RT3). The results showed that the conjugates could achieve an approximately 15-times higher toxicity effect on human breast cancer cells than free CPT in dimethyl sulfoxide, with similar enhancement of cytotoxic activity observed on skin tumor cells. Similarly, Zhong et al. [16] exploited π–π stacking interactions with a tri-block copolymer to prepare CPT-loaded MWCNTs. In their study, the MWCNTs were coated with the tri-block copolymer pluronic P123 to improve the solubility and anticancer effect of CPT. The resulting polymer-coated MWCNTs effectively formed non-covalent supramolecular complexes with CPT, and in vitro cytotoxicity tests using HeLa cells showed that the complexes had improved anticancer effects compared to free CPT. These results suggest that functionalized MWCNTs can improve the uptake of anticancer drugs in cancer cells, which is possibly attributed to the high aspect ratio and high surface area of the CNTs.

In an alternative approach, Yang et al. [65] loaded the anticancer molecule gemcitabine (GEM) into magnetic MWCNTs to inhibit cancer metastasis in the lymphatic system under magnetic guidance. To investigate the suppressive activity on cancer metastasis to the lymph nodes, the authors tested whether their magnetic MWCNTs could support the high loading capability of GEM with suitable in vitro cytotoxicity to cancer cells. The in vitro cytotoxicity results indicated that these complexes showed an approximately 62% loading efficiency with high cytotoxic activity against human pancreatic cancer cells (SW1990 and BxPC-3 cells). In addition, an in vivo study using a metastatic nude mouse model of pancreatic cancer demonstrated that these complexes could be delivered to the targeted site and successfully inhibited metastasis to the lymph nodes under magnetic guidance.

In another study, Tripisciano et al. [17] utilized purified and opened MWCNTs to load a high amount of irinotecan, a semisynthetic CPT analog with enhanced water solubility, and achieved a loading efficiency of ~32%. In addition to the fact that the irinotecan molecules were not degraded during this filling process, fast and complete release was observed in a weakly acidic environment (pH ≈ 6.0) due to the stability and hydrophilicity of irinotecan, which were increased in an acidic condition. Therefore, this finding suggests that the pH-dependent behaviors of MWCNTs loaded with irinotecan can be further applicable to colorectal cancer treatment, possibly via their pH-sensitive drug release activities.

**Figure 3:**
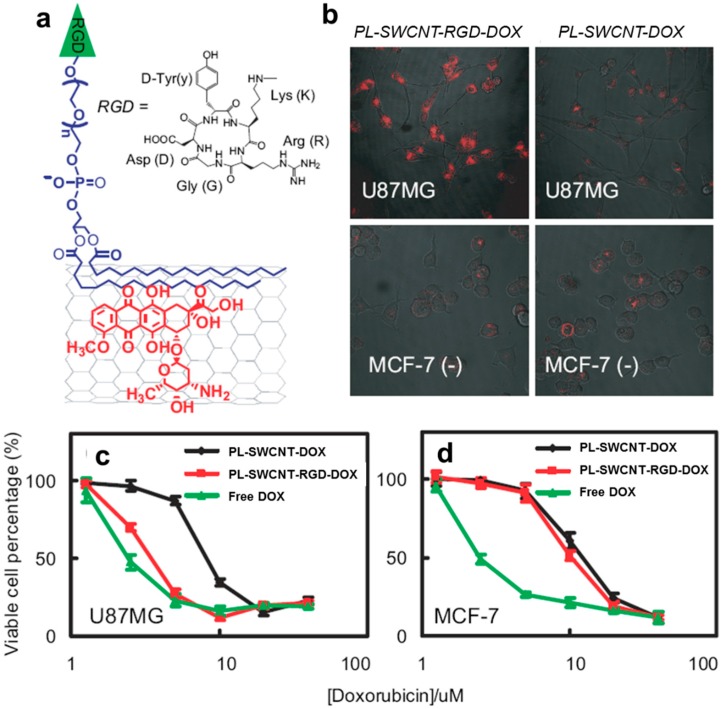
(**a**) Conjugation of cyclic arginine-glycine-aspartic acid (cRGD) peptide into PEG-functionalized SWCNTs (PL-SWCNT-RGD); (**b**) Immunofluorescence images of integrin α_v_β_3_-positive U87MG cells (top) and integrin α_v_β_3_-negative MCF-7 cells (bottom) with either the selective intracellular uptake of doxorubicin (DOX) via cRGD (top left) or non-specific delivery of DOX (the rest); (**c**,**d**) DOX dosage-dependent survival rates of U87MG cells (**c**) and MCF-7 cells (**d**) treated with various delivery vehicles as indicated. Reprinted with permission from Ref. [19]. Copyright (2016) American Chemical Society.

Currently, doxorubicin (DOX) is the most widely used anticancer chemotherapeutic agent, and a huge number of studies conducted in recent years have vigorously investigated the potential of CNTs as novel carriers for DOX. For example, Ali-Boucetta et al. [18] adopted a strategy to form supramolecular complexes of SWCNTs/MWCNTs with an aromatic chromophore and DOX through π–π stacking interactions. Their complexes showed enhanced cytotoxic activity to human breast cancer cells (MCF-7), whereas DOX-free MWCNT carriers did not show any cytotoxic effects. These results indicate that the inherent toxicity of MWCNTs did not have any detrimental effect on the viability of cancer cells, suggesting that MWCNT-based DOX delivery would facilitate the efficient delivery of water-insoluble anticancer drugs. Liu et al. [19] also developed a novel drug delivery system that consisted of polyethylene glycol (PEG)-functionalized SWCNTs, which allowed for strikingly higher degrees of π-stacking of an anticancer agent (DOX) with an extremely high loading capacity of ~400% by weight due to their high surface area. Furthermore, the authors demonstrated that conjugation of cyclic arginine-glycine-aspartic acid (cRGD) on PEG-functionalized SWCNTs loaded with DOX (PL-SWCNT-RGD-DOX) could recognize and selectively deliver DOX to integrin α_v_β_3_-positive human glioblastoma cancer cells (U87MG) intracellularly to induce apoptosis and cell death. By contrast, PL-SWCNT-RGD-DOX did not enhance DOX delivery to the α_v_β_3_-negative human breast cancer cells (MCF-7) (Figure 3).

Similarly, Datir et al. [66] created MWCNTs-hyaluronic acid (MWCNT-HA) conjugated to DOX by π–π interactions as a potential cancer-targeted drug delivery vehicle. The authors demonstrated that hyaluronan receptors (HRs) expressed on human lung adenocarcinoma cells (A549 cells) could internalize the MWCNT-HA by endocytosis, and the internalized MWCNT-HA showed lysosomal trafficking, resulting in 3.2-times higher cytotoxicity of the DOX-loaded HA-MWCNTs than free DOX at the same dose. In addition, the tumor-specific localization of the radiotracer technetium-99m (99mTc)-MWCNT-HA-DOX was improved compared to that of the free drug and the non-targeting MWCNTs in tumor-bearing mice. In animal models with induced breast cancer, injection of HA-MWCNT-DOX showed a more than 5-fold improvement in the inhibition of tumor growth compared to free DOX.

Similar approaches have been applied for the development of a targeted DOX delivery system using the cell surface receptor folate receptor (FR). For instance, Dinan and coworkers [20] synthesized folate-targeted PEGylated CNTs loaded with DOX via the conjugation of folate into the PEGylated CNT for DOX delivery. Their results demonstrated that the loading efficiency for DOX was decreased when using CNTs with higher levels of PEGylation, due to the increased hydrophilicity. However, faster release rates of DOX were observed for these higher PEGylated CNTs owing to the low affinity of DOX to the CNTs. Due to the presence of folate on PEGylated CNTs loaded with DOX, their drug vehicle could efficiently deliver DOX to the intracellular membrane of FR-positive human cervical cancer cells (HeLa cells). Therefore, their results suggest that the delivery of DOX can be effectively controlled simply by tuning the PEGylation level of CNTs. Zhang and co-workers [21] also developed a targeted delivery system based on folic acid (FA)-tethered SWCNT-DOX. Their results clearly demonstrated that FA-tethering could improve the targeting capability of the DOX-loaded SWCNTs. In addition to the cancer-targeting property, chitosan and sodium alginate were also introduced to improve their aqueous dispersion. After being internalized into HeLa cells by endocytosis, the drug complex could only be released into the cell in response to the low pH of the lysosome, and not under physiological condition (pH = 7.4). Li et al. [22] also designed a new delivery system by conjugating MWCNTs with iron NPs and FA. The authors demonstrated that this system supported a higher DOX loading efficiency and showed superior ability to deliver DOX into HeLa cells, which have FA receptors on the cell surface. Shi and co-workers [67] developed a novel method by covalently attaching poly(amidoamine) dendrimers to FA-treated MWCNTs for cancer targeting and the visualization of cancer cells. The use of these dendrimer-functionalized MWCNTs as drug delivery vehicles enabled the targeting of cancer cells that specifically overexpress FA receptors.

Recently, researchers have attempted to incorporate DOX-loaded CNTs into three-dimensional (3D) scaffolds to prolong the drug release profiles for local cancer treatment. Yu and coworkers [68] developed composite nanofibers using DOX-loaded CNTs and poly(lactic-*co*-glycolic acid) via electrospinning. DOX-loaded nanofibrous mat-type scaffolds were successfully fabricated through the electrospinning technique, and these scaffolds were able to control their release profiles over longer periods of time. The in vitro anticancer activity of these scaffolds was evaluated using HeLa cells, and the results showed selective cytotoxicity toward the cancer cells. These results demonstrate an alternative application of DOX-loaded scaffolds for treating local tumor sites.

In addition to the aforementioned DOX-based cancer therapeutics, studies have demonstrated the potential use of CNT-based delivery systems for other anticancer drugs. For example, Dhar et al. [23] developed amine-functionalized SWCNTs (SWCNT-PL-PEG-NH_2_) as a so-called “longboat delivery system” to effectively deliver the platinum-based anticancer drug cisplatin (*cis*-dichlorodiammineplatinum(II), CDDP) to tumor cells intracellularly. A complex of cisplatin and a folate derivative was linked to functionalize the SWCNTs by multiple amide bonds to comprise “longboats” that were ultimately endocytosed by FR-rich cancer cells, in which the drug was released and interacted with the nucleus to induce cellular apoptosis. Similarly, Tripisciano et al. [69] encapsulated cisplatin in SWCNTs instead of MWCNTs using a wet chemical approach with dimethylformamide. The authors demonstrated that the loaded cisplatin within SWCNTs was slowly discharged and reached its maximum release percentage (~68%) at 72 h. Their results further indicated that the CDDP-loaded SWCNTs inhibited the growth of prostate cancer cell lines (PC3 and DU145). In another study, Hampel et al. [24] incorporated a different platinum anticancer agent, carboplatin (*cis*-diammine(1,1-cyclobutanedicarboxylato)platinum(II); CP) into MWCNTs. The resulting CNTs were opened, and then a wet chemical approach based on the capillary force was used to drive CP into their interiors. Incorporation of CP into the MWCNTs was confirmed using electron energy-loss spectroscopy and X-ray photoelectron spectroscopy. in vitro studies demonstrated that the CP-loaded MWCNTs effectively inhibited the growth of urinary bladder cancer cells, whereas unfilled MWCNTs had little effect on cancer cell growth.

Since paclitaxel (PTX) is a well-known poorly water-soluble anticancer molecule, Cremophor EL (CrEL) is commonly used to dissolve the commercialized paclitaxel product (Taxol^®^). However, owing to the toxic nature of CrEL, several studies have been conducted to find an alternative solution to dissolve PTX [70]. In fact, the actual circulation time of Taxol^®^ is very limited, and therefore nano-carriers, including liposomes, are usually coated with hydrophilic polymers to prolong the circulation of the nano-carrier-entrapped molecules in the bloodstream by protecting the carrier from uptake by blood macrophages [13]. Indeed, the circulation time of PTX was shown to be increased by conjugation with PEG (PEGylation) [71]. Therefore, incorporation of branched PEG chains into the chemically functionalized SWCNTs conjugated with PTX provided the possibility of achieving tumor-targeted accumulation with minimal toxicity [25]. These PTX-SWCNTs showed a higher efficacy in suppressing tumor growth than clinical Taxol^®^, which was demonstrated in a murine 4T1 breast cancer model. The increased blood circulation of PTX-SWCNTs resulted in an approximately 10-times higher PTX uptake into the tumor. Lay et al. [26] adopted similar approaches to enhance anticancer drug activity by prolonging the circulation time. Specifically, they demonstrated that PEGylated SWCNTs or MWCNTs physically loaded with PTX by simply immersing the CNTs into a high-concentrated PTX solution could maintain the homogeneous PTX mixtures within the solution, and the PTX-loaded CNTs were able to suppress the growth of MCF-7 cancer cells and HeLa cells.

It is well established that multi-drug resistance is a major obstacle hindering the success of anticancer drug-based cancer therapy. Since the P-glycoprotein (P-gp) efflux transporter can disturb the accumulation of anticancer drugs in cancer cells, it is consequently a major contributor to reducing the effectiveness of cancer therapy [72,73]. For example, Cheng et al. [74] evaluated the potential use of PEGylated MWCNTs as novel drug carriers to reduce the development of multi-drug resistance. The authors demonstrated that the PEGylated MWCNTs could enter mammalian cells without causing any harm to the plasma membrane, and the resulting accumulation of PEGylated MWCNTs barely affected the cell cycle distribution or cell proliferation. PEGylated MWCNTs accumulated in the multidrug-resistant cell line HepG2-DR with the same efficiency as observed in the drug-sensitive cell line HepG2. All of these results clearly demonstrated that one of the main advantages of CNTs is to effectively deliver therapeutic chemicals directly to cancer cells.

### 2.2. CNTs as Carriers for Gene Therapy

The aim of gene therapy is to treat genetic disorders in diseased cells by correcting the genetic defect that causes the disease. The success of gene therapy with the most recent generation of disease-modifying medical interventions has been recognized with good therapeutic outcomes, offering a wide range of biologically active nucleic acids, which have been utilized to control the post-transcriptional or translational regulation of gene expression [75]. In gene therapy, the most important and difficult challenges are to deliver genes to the targeted cells by breaking through the cellular barriers. This task is particularly challenging because of the hydrophilic nature and large molecular size of genes. Therefore, vectors (viral or non-viral) must be used to deliver the genes and to target them intracellularly. Although non-viral vectors, including CNTs, are generally less efficient than viral vectors [76] and their life cycle is shorter than that of viral vectors, non-viral vectors are usually considered as relatively safe and can deliver genes without size limitations [77,78,79]. In this section, we focus on the potential applications of CNT-based vectors in gene therapy to treat cancer.

There have been numerous studies conducted to establish methods for efficiently delivering plasmid DNA to cells of interest [80]. For instance, Singh et al. [27] studied the interactions of three types of functionalized CNTs (f-CNTs) with plasmid DNA: ammonium-functionalized multi-walled and single-walled carbon nanotubes (MWCNTs-NH_3_^+^ and SWCNTs-NH_3_^+^), and lysine-functionalized single-walled carbon nanotubes (SWCNTs-Lys-NH_3_^+^). All of these cationic CNTs could condense DNA to various degrees. By controlling the crucial parameters such as the surface area and charge density of CNTs, the interaction and electrostatic complex formation between f-CNTs and plasmid DNA were demonstrated to be easily tunable. The authors also tested the ability of all three f-CNTs to deliver plasmid DNA to human lung carcinoma cells, and found that the f-CNT-associated delivery of plasmid DNA could induce higher gene expression levels of markers than naked DNA.

Similarly, Geyik et al. [28] transfected plasmid DNA using carboxylated MWCNTs. In their study, after the amino-modified linear plasmid DNA and the carboxylated MWCNTs were covalently bonded, the resulting complexes were successfully transformed into competent *Escherichia coli* cells in the absence of a heat-shock step at 42 °C, which is usually required for regular transformation processes. Their results confirmed the further enhancement in the delivery of linear DNA fragments. Recently, Liu and coworkers [29] revealed that the use of ammonium group-functionalized MWCNTs could enhance the transfection and expression efficiency of plasmid DNA in Ctenopharyngodon idellus kidney (CIK) cells. Both scanning electron microscopy and transmission electron microscopy showed a great affinity of CIK cells for MWCNTs-NH_3_^+^ and the good penetration of MWCNTs-NH_3_^+^ into the cellular membranes, respectively. The authors also performed polymerase chain reaction analysis to confirm that the MWCNTs-NH_3_^+^: DNA complexes were able to transfect CIK cells more effectively than naked DNA.

In another study, Karmakar et al. [30] developed ethylenediamine-functionalized SWCNTs, which were conjugated with the oncogene suppressor p53 gene (f-SWCNTs-p53). in vitro tests using MCF-7 breast cancer cells as a model system were conducted with an f-SWCNTs-p53 concentration of 20 µg/mL to evaluate whether the complexes could kill the cancer cells. After 72 h, approximately 40% of the cells exposed to f-SWCNTs-p53 underwent apoptosis, which was a far greater proportion than observed for cells exposed to either p53 or f-SWCNTs alone. In addition to the intracellular uptake of genes in cancer cells, caspase 3 activity, which is a well-known marker of induced apoptosis, was highly increased in cells incubated with the f-SWCNTs-p53 complex.

Pantarotto et al. [31] developed ammonium-f-SWCNTs associated with plasmid DNA. This complex could penetrate the membranes of mammalian cells and was able to stably remain inside the cells. The ammonium-f-SWCNTs showed low cytotoxicity and were able to deliver plasmid DNA to the cells efficiently. As a result, f-CNT-based DNA delivery could up-regulate gene expression by more than 10 times than that achieved using DNA delivery without f-CNTs. In another study, Gao et al. [32] demonstrated that MWCNTs functionalized with an amino group (NH_2_-MWCNTs) could bind to plasmid DNA by an electrostatic interaction and were able to transport it into cells. These results clearly demonstrate the potential of CNTs as plasmid DNA delivery systems for various therapies.

Recently, gene-silencing tools such as small interfering RNA (siRNA) and microRNA (miRNA) have been tested for their potential application in gene therapy. These approaches have increased the efficiency of treatment for various diseases, including cancers. siRNA and miRNA can be conjugated to functionalized CNTs using a disulfide linker, and then f-CNT complexes can induce the silencing and death of the targeted cells [81].

siRNA interferes with the expression of specific genes with complementary nucleotide sequences; this characteristic has been exploited to interfere with specific genes that are important in cancer propagation. Wang et al. [33] prepared f-SWCNTs to carry siRNA into K562 cells to inhibit the production of cyclinA2, which is important in cancer growth; the suppression of cyclinA2 expression by siRNA-CNTs inhibited cell proliferation and promoted apoptosis in the targeted tumor. Moreover, amino-functionalized siRNA-MWCNT complexes-mediated delivery of siRNA could lead to a decrease in the tumor mass, which resulted in the increased survival rate of animals afflicted with lung tumors [34]. Zhang et al. [35] conjugated functionalized SWCNTs with telomerase reverse transcriptase (TERT) siRNA to suppress the expression of the targeted gene expression, TERT, resulting in the inhibition of cancer cell growth as well as tumor growth. Complexes of cationically functionalized CNTs with polyethylene imines and siRNA achieved gene-silencing activity of up to 30% and cytotoxicity of up to 60% [36]. Bartholomeusz et al. [37] developed complexes of unmodified siRNA and pristine SWCNTs by utilizing simple sonication methods. The authors conjugated SWCNTs to siRNA to target the expression of hypoxia-inducible factor 1 alpha (HIF-1α), which is found in many human cancers and is associated with resistance to cancer therapy. The biological responses of these siRNA-SWCNT complexes were investigated using various types of cancer cells, and the results showed that the complexes strongly inhibited cellular HIF-1α activity. In tumor-bearing model mice, HIF-1α-siRNA-SWCNT complexes also effectively impeded the tumor HIF-1α activity. Recently, another interesting approach utilizing functionalized MWCNTs (f-MWCNTs) to deliver siRNA for lung cancer therapy was presented by Guo and colleagues [38]. The authors demonstrated that cationic MWCNT-NH_3_^+^ was able to efficiently deliver the apoptotic siRNA against polo-like kinase (PLK1) in human lung carcinoma xenografts. Their results revealed that injections of their siPLK1-MWCNT-NH_3_^+^ complexes intratumorally could significantly induce apoptosis of tumor cells and therefore prolong animal survival rates. In addition, for the first time, their findings indicated that therapeutic efficacy achieved by f-MWCNT-mediated siRNA delivery was directly proportional to the siRNA retention in the tumor mass.

Since miRNAs are small non-coding RNA molecules that function in RNA silencing and the post-transcriptional regulation of gene expression, they have also been combined with CNTs to combat cancer. For example, Dong et al. [82] proposed a polyethylenimine-grafted graphene nanoribbon (PEI-g-GNR) as a novel carrier of miRNA. The PEI-g-GNR was prepared to protect locked nucleic acid-modified molecular beacon (LNA-m-MB) probes from interaction of single-stranded binding proteins or digestion by nucleases. They successfully demonstrated that the PEI-g-GNR had negligible cytotoxicity and induced minimal apoptosis under the optimized transfection condition. The combination of LNA and miRNA in the LNA-m-MB/PEI-g-GNR delivery system could efficiently transfer LNA-m-MB into cells, which was capable of then recognizing the target miRNAs. Therefore, these results suggest that PEI-g-GNR is a promising candidate for use in gene therapy.

**Figure 4:**
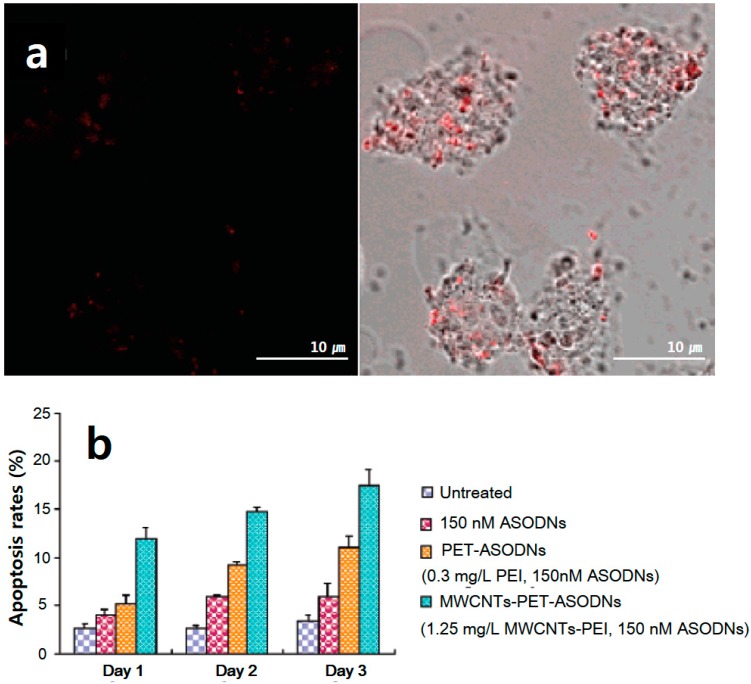
(**a**) Confocal fluorescence image of HeLa cells taken 24 h after 1 h-long incubation with MWCNTs-PEI-ASODNs-CdTe at 37 °C (left), and the merged image (right); (**b**) Flow cytometric analysis. After culturing the cells in the presence of naked ASODNs, PEI-ASODNs, and MWCNTs-PEI-ASODNs, cell viabilities up to day 3 were analyzed by staining the cells with propidium iodide (PI). Reprinted with permission from Ref. [83]. Copyright (2016) American Chemical Society.

Antisense therapy is another important technique to cure tumors or genetic disorders. Antisense oligodeoxynucleotides (ASODNs) can bind to the start location of mRNA translation, thereby blocking the translation of target mRNA into protein, consequently inhibiting target gene expression at the protein level [39,83]. Pan et al. [39] conjugated ASODNs with polyamidoamine dendrimer-modified MWCNTs (dMWCNTs) to improve the efficiency of gene delivery. These composites hindered the growth of human breast cancer cells (MCF-7 and MDA-MB-435) and liver cancer cells (HepG2), and down-regulated the expression of the C-Myc gene and C-Myc protein. Jia et al. [83] studied a double-functionalized CNT-based delivery system, using ASODNs as a therapeutic target gene (with cytotoxic activity) and semiconductor quantum dots (CdTe) as fluorescent probes (for imaging) (Figure 4). These composites were endocytosed by human cervical cancer (HeLa) cells and the ODNs were delivered intracellularly, leading to strong nuclear localization and extensive apoptosis.

Aptamers are RNA- or DNA-based single-stranded short oligonucleotides that can use shape matching to recognize their intracellular targets. The strategy of using CNTs to deliver DNA/RNA aptamers has been widely evaluated as a potential gene therapy technique. Because they are capable of disrupting protein–protein interactions, they can be utilized to inhibit intracellular pathways, and have therefore been recognized for their various potential therapeutic applications [84,85]. For instance, Bossche et al. [40] grafted aptamers onto carboxylated CNTs to use them as a vector system that can be easily translocated into the cytosol of different cell types, independent of receptor-mediated uptake. Efficient intracellular delivery was achieved with these apatamer-based composites, showing the promising potential of biologically active aptamers for therapeutic applications.

### 2.3. CNTs as Carriers of Other Compounds

Immunotherapy is another possible approach for treating incurable cancer. Along with the recent extension of immunotherapy to cancer therapy, anticancer immunotherapy using CNTs has been heavily investigated because of their good potential in targeting tumors. McDevitt and co-workers [86] attached tumor-specific monoclonal antibodies to SWCNTs to target lymphoma, and used a metal-ion chelate to carry or deliver a radioactive metal ion and fluorescent chromophores to visualize their location. In addition, Ruggiero et al. [41] attached both tumor-associated angiogenesis-targeting antibodies E4G10 and radioactive metal ion chelates to SWCNTs. Owing to their dual functions in targeting and radioimmunotherapy, this formulation could simultaneously reduce the tumor volume and extend the survival rate of LS174T tumor-bearing model mice. In another study, Meng et al. [42] investigated the immunological responses that were induced by oxidized MWCNTs in a subcutaneous injection model of hepatocarcinoma-bearing mice. The injected CNTs induced significant increases in the production of inflammatory cytokines and stimulation of phagocytosis by macrophages. These responses promoted the activity of the host immune system and inhibited tumor growth. Therefore, these results suggest that CNTs can induce anticancer activity by provoking a host immune response against tumors.

Glioma (brain tumor) cells secrete immunosuppressive cytokines such as transforming growth factor-beta, prostaglandin E, and interleukin (IL)-10, and are also highly capable of evading the host immune system [87,88]. Therefore, successful treatment of glioma has thus far not been possible with conventional chemotherapy. To overcome this challenge, Van Handel et al. [43] proposed an immunotherapeutic approach using MWCNTs based on the preferential uptake of CNTs by macrophages compared to glioma cells. Their results showed that the injection of MWCNTs increased the influx of macrophages into the glioma cells and caused an increase of the tumor cytokine level (IL-10) in time-dependent and dose-dependent manners. Moreover, the injected MWCNTs did not show notable toxicity in normal or tumor-bearing mice. These results suggest that immune modulation through CNTs is a promising strategy for brain tumor therapy.

Ouyang and co-workers [44] assessed the effects of SWCNT-CpG (Cytosine-Phosphate-Guanine) complexes under clinically relevant situations using an animal model of invasive glioma (K-Luc). In their study, by combining SWCNT-CpG with temozolomide, the standard-of-care chemotherapy for glioblastoma patients, they demonstrated that the SWCNT-CpG was well tolerated and prolonged the survival of glioma-bearing mice. In addition, the anti-tumor capacity of SWCNT-CpG was improved in combination with temozolomide. These results demonstrated that the utilization of SWCNTs with immunotherapy could be a promising approach for treating devastating glioblastomas. In another recent study, Fadel et al. [45] developed a polymer-CNT complex, which functions as an antigen-presenting cell (APC) to promote the proliferation of T cells. This could be achieved by attaching specific antigens into the bundled CNTs, and these complexes were then combined with polymeric NPs containing magnetite and the T-cell growth factor IL-2. Their results demonstrated that a 1000-fold reduction in the amount of IL-2 loaded in their CNTs could exert the same function as achieved with the clinically available standards of IL-2. Furthermore, using in vivo melanoma experiments, the authors successfully showed that their artificial APCs could promote the growth of T cells, which suppressed the growth of tumors in an animal model. Thus, their results indicate the potential application of CNTs for cancer therapy using immune cells.

CNTs have also been applied to deliver various proteins with anticancer activity, such as streptavidin, a protein purified from the bacterium *Streptomyces avidinii* [89]. Since the large molecular weight (~60 kDa) of streptavidin hinders its ability to penetrate cells, Kam et al. [46] conjugated streptavidin into an SWCNT-biotin transporter to efficiently internalize streptavidin into promyelocytic leukemia (HL60) cells and human T cells (Jurkat) by exploiting an endocytosis pathway. The uptake pathway of the conjugates was consistent with a mechanism of endocytosis, and these results suggested that SWCNTs have possible applications as transporters for various cargos.

Weng et al. [47] studied the recombined protein toxin (RTA)-induced targeted destruction of tumor cells via CNT transporters. Conjugates of MWCNT and RTA were internalized into various cell lines and effectively induced cell death. The cell death rates of HL7702, L-929, HeLa, MCF-7, and COS-7 cells caused by RTA-MWCNTs conjugates were three-times higher than those caused by RTA alone; for example, 75% of the HeLa cells died after treatment with RTA-MWCNTs. These results suggest that CNTs as transporters of functional proteins may provide new methods of cancer therapy.

## 3. CNTs as Imaging Sources for Cancer Detection

CNTs have been widely used in bioimaging because of their unique physical properties. SWCNTs have a narrow band gap of ~1 eV, so that NIR (wavelength 700 ≤ λ ≤ 1400 nm) fluorescence emission is possible [90,91,92]. SWCNTs also have the ability to show strong resonance of Raman scattering, indicating their utility as Raman probes for biological sensing and imaging [93,94]. In addition, CNTs strongly absorb in the NIR region, making them useful contrast agents in PA imaging [95,96]. Indeed, incorporation of metal NPs in impure CNTs facilitates MRI by supplying imaging contrast [97]. In this section, we summarize the latest noteworthy results of various CNT-based imaging methods.

### 3.1. CNTs for Fluorescence Imaging

Fluorescence imaging is an important biomedical imaging tool, but the low penetration depth of visible light into tissues has thus far hindered its practical use [98]. As a solution to overcome this limitation, researchers have developed fluorescent probes that have emission/excitation wavelengths in the NIR spectrum, in which biological tissues are transparent [99]. Various organic dyes [100,101,102] and QDs [103] have been developed to improve the tissue penetration and spatial resolution of in vitro and in vivo fluorescence imaging [90,104,105,106,107]. Among the various candidates, the unique physical properties of CNTs make them the best candidates as fluorescence probes [96,108]. SWCNTs are quasi one-dimensional wires; they have a narrow band gap of ~1 eV, which provides them with semiconducting properties. Therefore, they allow for NIR-II (1000 ≤ *λ* ≤ 1700 nm) fluorescence under excitation in the NIR-I window (750 ≤ *λ* ≤ 900 nm) [109]. Moreover, their low auto-fluorescence prevents the degradation of the sensitivity during in vivo imaging [48,92].

There have been attempts to develop biocompatible SWCNTs with a high quantum yield. For example, Welsher et al. [48] used a gentle method of the step sonication of SWCNTs with sodium cholate and PEG, which did not cause severe damage to the CNTs during the processes. Given that NIR can penetrate into deep tissue with a low auto-fluorescence background, the use of the CNTs as contrast enhancers enabled the high-resolution microscopy imaging of tumor vessels underneath thick skin. In addition, to investigate the mouse anatomy, they performed fluorescence video imaging of the mouse with a high-frame rate during injection of SWCNTs as an NIR-II contrast agent. The authors observed the circulation of SWCNTs in real time through the lungs, kidneys, spleen, and liver, and their dynamic contrast imaging results analyzed with principal components analysis demonstrated great improvement in the anatomical resolution of the organs [48]. Thus, their study confirmed the usefulness of NIR-II fluorescence imaging using CNTs.

Similarly, Robinson et al. [49] developed well-functionalized SWCNTs that included poly(maleic anhydride-alt-1-octadecene)-PEG, which showed long blood circulation (half-life of 30 h) in Balb/c mice bearing 4T1 murine breast cancer tumors, with high accumulation of a ~30% injected dose (30% ID/g). By exploiting the fluorescence of SWCNTs in the NIR-II window and dynamic contrast imaging using principal components analysis, they were able to capture video-rate imaging of tumors within ~20 s after injection. In addition, their SWCNTs photoluminescence-based 3D reconstruction results showed enhanced permeability and retention due to the high level of uptake of the nanotubes by the tumor. The authors also performed in vivo real-time fluorescence video imaging of the mouse hind limb vasculatures, using SWCNTs as fluorophores in the NIR-II region. These results further confirmed the superiority of this method compared to ultrasonography at low blood velocities.

Given the great advantages of SWCNT photoluminescence-based imaging technology, Hong et al. [110] further demonstrated that their SWCNT-based NIR-II imaging methods could achieve higher spatial resolution and speed acquisition than are possible with conventional in vivo microscopic-computed tomography. Furthermore, by extending their findings toward developing a novel through-skull fluorescence imaging technology, they achieved the non-invasive high-resolution imaging of the outside layer of the mouse brain [111]. The authors utilized the intrinsic photoluminescence of SWCNTs in the narrow 1300–1400 nm region, known as the NIR-IIa region, and found reduced photon scattering in this region, allowing them to acquire fluorescence images over a depth of 2 mm with a resolution below 10 μm. These novel methods allowed for the dynamic recording of blood perfusion in the cerebral vessels and the real-time assessment of inharmonic blood flow in a mouse artery occlusion stroke model.

Although many studies have used SWCNT-based contrast agents for the NIR-IIa region (1300–1400 nm), Diao et al. [112] recently extended these applications by investigating the capability of semi-conducting SWCNTs for in vivo fluorescence-based imaging of live animals at the long-wavelength NIR-IIb region (1500–1700 nm). By employing laser vaporization techniques, the authors were able to identify the optimal diameter of SWCNTs, with an average diameter of 0.9–1.4 nm, and the resulting SWCNTs yielded the brightest fluorescence in the NIR-IIb region due to the smaller band gaps and larger diameter. Since their SWCNTs with the optimal diameter were found to minimize the scattering of photons and absorption of water in living tissues, the authors have demonstrated their potential as non-invasive in vivo fluorescence imaging modalities that can penetrate deep tissue with high resolution. For example, when they exploited the SWCNTs as imaging agents, they were able to obtain high-magnification vascular images of the mouse hind limb and brain with a spatial resolution of 3–4 μm at the deep-tissue level (approximately 3 mm). The findings from this study using semi-conducting SWCNTs with larger diameters have shed light into the future of NIR-IIb imaging applications.

In another study, Ghosh et al. [50] developed M13 virus-stabilized SWCNTs to detect propagated tumors within host tissues. After the M13 viruses were bound and stabilized to SWCNTs, the authors achieved the targeted imaging of tumors, thereby confirming that the M13-SWCNTs could function as SWCNT imaging probes with high signal-to-noise performance and high tumor-to-background uptake. By leveraging the SWCNTs’ image guidance to minimize the auto-fluorescence and tissue scattering in the NIR-II region, ovarian tumors were precisely identified at sub-millimeter resolution and were surgically removed. These findings validated the potential for the targeted imaging of tumors via M13-SWCNTs for the dual function of noninvasive disease monitoring and image-guided surgery.

**Figure 5:**
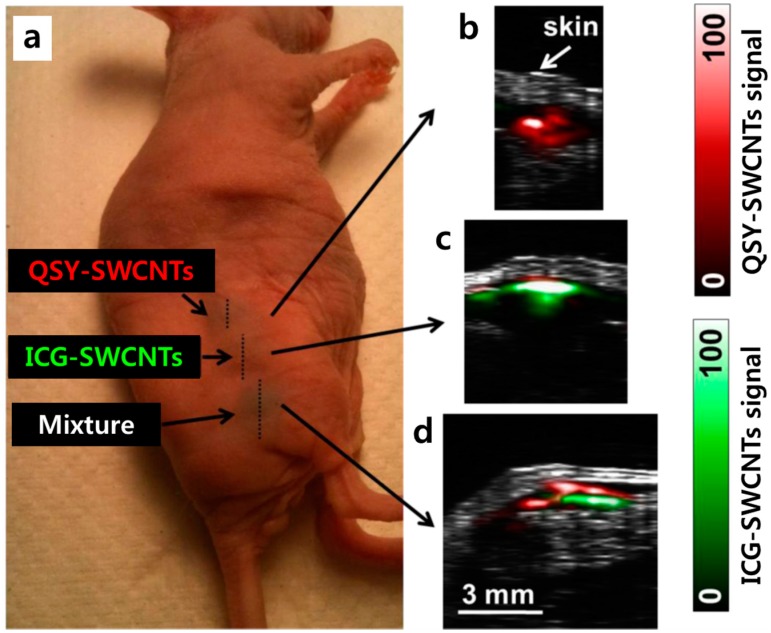
(**a**) A mouse was injected with 30 μL of 50 nM QSY-SWCNTs (upper inclusion), 30 μL of 50 nM ICG-SWCNTs (middle inclusion), and 30 μL of an equal mixture of 50 nM of QSY-SWCNTs and ICG-SWCNTs (lower inclusion); (**b**) The unmixed photoacoustic vertical slice through the upper inclusion, showing only the QSY-SWCNTs signal (red); (**c**) The unmixed photoacoustic slice through the middle inclusion showing mostly the ICG-SWCNTs signal (green); (**d**) The unmixed photoacoustic slice through the lower inclusion, showing both the QSY-SWCNTs and ICG-SWCNTs signals spread throughout the inclusion area. The segregation between the QSY-SWCNTs and ICG-SWCNTs signals occurred because of some artifacts during the image reconstruction process due to the movement of the animal and heterogeneous penetration of light. Reprinted with permission from Reference [56]. Copyright (2016) American Chemical Society.

In an effort to achieve enhanced visualization of tumors and higher efficiency for the photothermal ablation of tumors, Antaris et al. [51] developed SWCNTs with bright photoluminescence by incorporating a biocompatible surfactant, C_18_-PMH-mPEG (poly(maleic anhydride-alt-1-octadecene)-methoxy poly(ethyleneglycol)). Indeed, the C_18_-PMH-mPEG-coated (6,5) SWCNTs developed in their study showed 6-fold brighter photoluminescence and provided distinct tumor imaging (Figure 5). Furthermore, they demonstrated that their materials could reach the requisite photothermal tumor ablation temperatures at a 10-fold lower dose than required with their counterparts (as-synthesized SWCNT mixtures). This suggests that a low dose (~4 μg) of (6,5) SWCNTs would be sufficient to support the dual imaging/photothermal functions to further utilize this material in SWCNT-based nanomedicine.

### 3.2. CNTs for Raman Imaging

Raman scattering is the phenomenon by which the energy exchange between a photon and molecule leads to inelastic scatter. In Raman scattering, the scattered photon usually has lower energy than the incident photon. However, when the energy from the outside matches the energy needed for the electron transition, the scattering efficiency increases; this phenomenon is called the resonance of Raman scattering [113]. The Raman spectrum of SWCNTs shows several scattering modes, including the radial breathing mode (RBM, 100–350 cm^−1^) due to carbon atom vibration in the radial direction, and the tangential G band (1580 cm^−1^) due to carbon atom vibration in the tangential direction. The intensity of the resonance Raman scattering by SWCNTs is determined by the state density based on their diameters and chirality indices [114].

Heller et al. [115,116] first exploited the Raman properties of SWCNTs to image live cells, opening the door for numerous studies to expand and optimize these applications. For example, Lamprecht et al. [52] focused on the Raman G band to study the targeted delivery of FA-functionalized double-walled CNTs (DWCNTs) to human urinary bladder carcinoma (T24) cells that overexpress an FA receptor. The intense G mode observed in the Raman spectra of internalized DWCNTs proved that the DWCNTs were localized inside the carcinoma cells. This method could also be used to map the locations of carcinoma cells and track their movements. Namely, Raman imaging using CNTs shows great potential for intracellular imaging. In another study, Zavaleta et al. [53] used an optimized Raman microscope to evaluate the tumor-targeting ability and localization of SWCNTs in mice. To improve the quality of the image, they used RGD-SWCNTs; the injected RGD-SWCNTs accumulated for >72 h in the tumor area. Their results showed the feasibility of SWCNTs to improve preclinical imaging.

Similarly, Smith et al. [54] used SWCNTs in high-resolution intravital microscopic imaging. They conjugated RGD peptide to SWCNTs to target the specific integrin α_v_β_3_, which is overexpressed in the tumor vasculature and on the surface of tumor cells (U87MG). Their results showed that binding of the RGD-SWCNTs to tumor cells was four-times higher than that of the control arginine-alanine-aspartic acid (RAD)-SWCNTs, and a significant amount of SWCNTs remained bound to the tumor cells even after four weeks. These results further demonstrate the potential applications of SWCNTs to target specific cancer cells. In another study, Liu et al. [55] synthesized SWCNTs with five different ^13^C/^12^C isotope compositions achieving well-separated Raman peaks, conjugated the SWCNTs to five targeting ligands to impart molecular specificity, and performed multiplexed live-cell Raman imaging by staining the cells with a five-color mixture of SWCNTs. The imaging results using tumor samples showed the significant up-regulation of epidermal growth factor receptor on LS174T colon cancer cells, and the fine resolution could be accomplished owing to the sharp Raman peaks of CNTs. These results indicate the feasibility of using SWCNTs as NIR Raman tags for multi-color optical imaging and detection purposes.

### 3.3. CNTs for Photoacoustic (PA) Imaging

PA imaging is a recently developed imaging method based on the following principle [117,118,119]. First, laser pulses are absorbed by endogenous molecules or contrast agents, which expand due to absorbed energy. This expansion causes wide-band ultrasonic emission, which can be detected by an ultrasound microphone. The data are processed to construct 2D or 3D images. Because PA imaging detects sound, it has several advantages, including good tissue penetration and fine spatial resolution. Various nanomaterials with strong NIR absorbance have been evaluated as potential contrast agents in PA imaging [120,121,122].

For example, de la Zerda et al. [123,124] introduced RGD-SWCNTs as PA contrast agents. The PA signals were stronger in tumor-bearing mice injected with RGD-SWCNTs than in those injected with plain SWCNTs. This result contributed to the discovery of a new tool for the non-invasive imaging of cancers. In their follow-up study, the authors [56] further synthesized SWCNT-dye PA contrast agents by coating SWCNTs with indocyanine green dye (ICG-SWCNTs). These ICG-SWCNTs were further conjugated with cyclic RGD peptides to function as tumor-targeted agents for detecting tumors in disease models. Intravenous (IV) injection of ICG-SWCNTs into the tumor-bearing mice showed a distinctly higher PA signal at the tumor sites compared to IV injection of the untargeted contrast agent in the same mice. Furthermore, their new agents showed sub-nanomolar sensitivity in living tissues and 20-times higher cancer cell detection than achieved with previously reported SWCNTs. As shown in Figure 5, QSY21 (QSY-SWCNTs) or ICG-SWCNTs showed strong absorbance spectra and were successfully utilized for multi-color PA imaging in vivo.

Galanzha et al. [57] demonstrated the use of rapid PA detection to search for circulating tumor cells in the mouse bloodstream under a magnetic field, and then used PA as a contrast agent to acquire images of the tumor-bearing mice. To improve the detection sensitivity and specificity of the imaging, they used a second contrast agent composed of gold-plated CNTs conjugated with FA. In a recent study, Nguyen and coworkers [58] validated that ICG is adsorbed onto the CNTs and SWCNT-ICG could improve the PA imaging sensitivity. In their experiment, the amplitudes of the PA signal were linearly increased whenever the ICG-SWCNT concentration was raised upon strong light absorption, and the signal intensity of their ICG-SWCNTs was 2-fold higher than that of the SWCNTs alone. Their approach shows potential of these complexes for the prevention of metastasis and the diagnosis of cancer.

Xie and coworkers [59] recently fabricated a long-term circulation SWCNT complex using a new dispersion agent, Evans blue (EB). Their complex was endowed with fluorescent imaging and photodynamic therapy ability by the self-assembled loading of an albumin-coupled fluorescent photosensitizer, chlorin e6 (Ce6), which shows high affinity to EB. Albumin/Ce6-loaded EB-CNTs provided the ability for the fluorescent and PA imaging of tumors. In addition, synergistic photodynamic therapy and photothermal therapy could be carried out as guided by the imaging results obtained at 24 h post-injection, and achieved an efficient tumor ablation effect. Their results demonstrated that EB can be extended to other nanomaterials to improve their in vivo stability and circulation time.

### 3.4. CNTs for Magnetic Resonance Imaging (MRI)

MRI is a particularly powerful technique because it can acquire a whole-body image without a depth limit [125]. Approximately 60 million people undergo MRI procedures annually, and contrast agents are used in ~30% of these procedures [126]. Contrast agents for MRI are classified into T_1_-shortening agents (containing transition metal ions such as Gd^3+^ and Mn^2+^) and T_2_-shortening agents (e.g., iron oxide NPs). It is well established that CNTs can be doped with metallic impurities during the synthesis processes, allowing them to be used as MRI contrast agents without requiring additional treatment. In fact, numerous studies have been conducted to date to evaluate the potential of using CNTs for this purpose [97,127,128,129,130,131].

In one such study, Sitharaman et al. [132] investigated the magnetic behavior, relaxometry, and phantom MRI of gadolinium-catalyzed SWCNTs (Gd-SWCNTs). The authors demonstrated that the T_1_-weighted MRI signal intensity of the Gd-SWCNT phantom solution was 14-times higher than that of the commercial Gd-based clinical MRI contrast agent (Magnevist). These results suggest that Gd-SWCNTs have potential as highly efficacious MRI-NIR imaging contrast agents. Similarly, Richard et al. [60] synthesized an amphiphilic gadolinium (III) chelate (GdL) using stearic acid. In their study, after stabilizing the aqueous GdL solutions with varying concentrations ranging from 1 mM to 1 μM, which were embedded into MWCNTs (MWCNTs/GdL), the authors evaluated their potential application as T_1_ and/or T_2_ MRI contrast agents. To this end, the functionalized nanotubes (MWCNTs/GdL) were intramuscularly injected into the mouse legs, and the results clearly demonstrated that MWCNTs/GdL could function as both positive and negative paramagnetic contrast agents.

In contrast to the aforementioned studies in which the MWCNTs were non-covalently functionalized with an amphiphilic GdL chelate, Marangon and coworkers [61] developed water-dispersible non-toxic CNTs, which were covalently functionalized with the chelating ligand diethylenetriaminepentaacetic dianhydride, following by the anchoring of gadolinium (Gd^3+^) ions to the MWCNTs (Gd-CNTs). Use of the Gd-CNT conjugates resulted in powerful T_1_ contrast MR signal enrichment upon cellular internalization, and their uptake into organs such as the liver and spleen was successfully demonstrated using in vivo MRI. Their results provide new possibilities for evaluating the biodistribution of functionalized CNTs and for developing a novel image-guided delivery modality.

Moreover, Choi et al. [133] reported T_2_-weighted MRI contrast agents that were hetero-structured complexes consisting of NIR fluorescent SWCNTs with magnetic iron oxide NPs. By varying the contents of iron oxide NPs, their complexes showed the distinct features of the different nanotube species in Raman scattering, visible/NIR absorbance, and NIR fluorescence. Superconducting quantum interference device and X-ray diffraction analyses revealed the superparamagnetic characteristics of the NPs. Because their magnetic components were small (~3 nm), they showed a longer spin-spin relaxation time (T_2_ ≈ 164 ms) compared to that of typical ferromagnetic particles. In addition, MRI revealed the uptake of the DNA-wrapped complex by cells, which demonstrated the potential applications of these multifunctional nanostructures for novel live-cell imaging techniques.

A similar approach was reported by Wu et al. [62] who synthesized MWCNTs/cobalt ferrite (CoFe_2_O_4_) magnetic hybrids with a solvothermal method. Their magnetic hybrids demonstrated a high T_2_ relaxivity of 152.8 Fe mM^−1^·s^−1^ in aqueous solutions, and exhibited an apparent negative contrast enhancement, low cytotoxic effect, and negligible hemolytic activity. In addition to their functions as MRI contrast agents, magnetic hybrids loaded with DOX showed a severe cytotoxic effect in HeLa cancer cells due to their ability to release DOX intracellularly. These results indicate that MWCNTs/cobalt ferrite hybrids have great potential as both MRI agents for effectively detecting cancers and as novel drug delivery systems for chemotherapy applications.

Peci and coworkers [63] developed functionalized CNTs that can be used as a contrast agent for MRI as well as a material for targeted cancer therapy via magnetic hyperthermia. In their study, they introduced CNTs with ferromagnetic properties by filling up a central capillary with iron NPs, and the sidewalls of these CNTs were functionalized with Gd^3+^ ions. Since the ferromagnetic properties of the encapsulated iron NPs at room temperature were maintained after surface functionalization, their Gd-doped CNTs fulfilled the clinical requirements for hyperthermia treatment. Therefore, their results suggest that these hybrid structures have great potential as candidates for MRI and magnetic hyperthermia cancer therapy.

## 4. Conclusions and Perspectives

Carbon nanotubes (CNTs) are attractive transporters for the delivery of biomolecules, drugs, and agents. Appropriate functionalization enables the use of CNTs as nano-carriers to transport anticancer drugs [134]. CNTs have also been used as carriers for genes, including siRNA, miRNA, ODNs, DNA, and RNA/DNA aptamers. CNTs can also deliver proteins and immunotherapy components. In cancer imaging studies, CNTs are the most extensively used materials to visualize targeted tumors owing to their ability of leveraging various imaging modalities such as (1) fluorescence imaging due to improved fluorescence in the near-infrared (NIR)-II biological transparent window; (2) Raman imaging due to the strong Raman scattering resonance; (3) photoacoustic (PA) imaging due to high ultrasonic emission as a result of strong NIR absorbance; and (4) magnetic resonance imaging (MRI) due to the inherent magnetic property of CNTs derived from their metallic impurities.

Despite the promising results of in vivo and in vitro studies evaluating the potential of CNT-based materials as therapeutic and imaging carriers, several concerns regarding their clinical applications are yet to be addressed [135,136]. First, although various functionalizations and chemical modifications of CNTs have successfully mitigated or bypassed their cytotoxic effects on numerous cell types and physiological systems, the broad safety issues and risk factors of using CNTs, including their toxicity, cytotoxicity, carcinogenicity, and genotoxicity, in the human body have to be fully assessed. In addition, since most of the in vivo toxicity tests reported to date were performed over relatively short periods of time, studies investigating the long-term toxicity of CNTs are now essential. Second, the targeting accuracy as well as the efficacy of a CNT-based drug delivery system should be further implemented by controlling the degradability of CNTs to maximize their drug loading/unloading efficiencies, stability of the drugs, and activity of the conjugated drugs within CNTs. Finally, the uniformity of CNTs and drug-CNT complexes must be achieved to ensure the delivery of the correct doses during actual treatment. If these issues currently facing CNT-based technologies are accurately resolved, the applications of CNTs will certainly be extended to various fields of biomedicine, particularly in improving chemotherapy and cancer diagnosis.

## Figures and Tables

**Table 1:** Applications of functionalized carbon nanotubes for the therapy and diagnosis of cancer in this review.

Application	Chemical Structure of Carbon Nanotubes (CNTs) (Functional Group)	Study Design (in vitro/in vivo)	Results	Reference
HCPT	f-MWCNTs (diaminotriethylene glycol spacers)	MKN-28 (in vitro)	Superior antitumor activity	[14]
		Hepatic H22 tumor bearing ICR mice (in vivo)		
CPT	f-MWCNTs (PVA)	MDA-MB23, A-5RT3 (in vitro)	15-fold higher mortality than free CPT	[15]
CPT	f-MWCNTs (Pluronic P123)	HeLa (in vitro)	Enhanced antitumor activity over free CPT	[16]
Irinotecan	MWCNTs (open tips)	-	Higher filling amount of Irinotecan at a larger inner diameter tube	[17]
DOX	f-MWCNTs (dispersed Pluronic F127)	MCF-7 (in vitro)	Enhanced cytotoxicity on human breast cancer cells	[18]
DOX	f-SWCNTs (PEG and cyclic RGD)	U87MG, MCF-7 (in vitro)	Extremely high loading efficiency of ~400%	[19]
DOX	f-MWCNTs (PEG)	HeLa (in vitro)	Higher cell killing effect	[20]
DOX	f-SWCNTs ((CHI and/or ALG) and FA)	HeLa (in vitro)	Release of DOX at low pH.	[21]
DOX	f-MWCNTs (FA and iron NP)	HeLa (in vitro)	6-fold higher delivering ability than free DOX	[22]
CDDP	SWCNTs	PC-3, DU145 (in vitro)	Smooth release profile until 72 h	[23]
CP	Oxidized MWCNTs (open tips)	EJ28 (in vitro)	Growth inhibition of bladder cancer cells	[24]
PTX	f-SWCNTs (PEG phospholipids)	4T1 (in vitro)	10-fold higher uptake than clinical Taxol^®^	[25]
		Mice baring 4T1 tumors (in vivo)		
PTX	f-SWCNTs and f-MWCNTs (PEG)	MCF-7, HeLa (in vitro)	Growth suppression of cancer cells	[26]
Plasmid DNA	f-SWCNT (–NH_3_^+^, Lys-NH_3_^+^)	A549 (in vitro)	Up-regulation of marker gene expression over naked DNA	[27]
	f-MWCNTs (-NH_3_^+^)			
Plasmid DNA	f-MWCNTs (–COOH)	Escherichia coli (in vitro)	Improvement for delivery of linear DNA fragments	[28]
Plasmid DNA	f-MWCNTs (–NH_3_^+^)	CIK (in vitro)	effective transfection in CIK cells than naked DNA	[29]
Plasmid DNA	f-SWCNTs (ethylenediamine)	MCF-7 (in vitro)	40% apoptosis after 72 h exposure	[30]
Plasmid DNA	f-SWCNTs and f-MWCNTs (NH_2_)	HeLa (in vitro)	Higher pDNA uptake and gene expression than pDNA alone	[31]
Plasmid DNA	f-MWCNTs (NH_2_)	A375 (in vitro)	Successful delivery of the GFP gene in cultured cells	[32]
siRNA	f-SWCNTs (NH_2_)	K562 (in vitro)	Inhibition of cell proliferation and promotion of cancer apoptosis	[33]
siRNA	f-MWCNTs (NH_3_^+^)	Calu6, SVEC 4–10 and 2F2B, DU145 and C-33A, A549, MCF7, HeLa, HEK293, B16F10, NIH 3T3 (in vitro)	Delay of the tumor growth (in vitro) Increased survival of xenograft-bearing animals (in vivo)	[34]
		Mice bearing Calu6 xenograft tumors (in vivo)		
siRNA	f-SWCNTs (–CONH-(CH_2_)_6_-NH_3_^+^Cl^−^)	TC-1 cells, 1H8 cells, LLC cells (in vitro)	Reduced tumor growth (in vivo)	[35]
		Mice bearing Lewis lung carcinoma or HeLa cell xenografts (in vivo)		
siRNA	f-MWCNTs (-PEI and -pyridinium)	H1299 (in vitro)	10%–30% silencing activity and a cytotoxicity of 10%–60%	[36]
siRNA	Pristine SWCNTs	MiaPaCa-2/HRE, MCF-7, MDA-MB-231, RGM1 (in vitro)	Significantly inhibition of Hif-1α expression (in vitro)	[37]
		Mice bearing MiaPaCa-2/HRE tumors (in vivo)		
siRNA	f-MWCNTs ((NH_3_^+^)	Calu6 (in vitro)	Reduced tumor growth (in vivo)	[38]
		Mice bearing Calu6 lung carcinoma (in vivo)		
ODNs	f-MWCNTs (polyamidoamine dendrimer modified)	MCF-7, MDA-MB-435, HepG2 (in vitro)	Inhibition of the cell growth (time- and dose-dependent)/Down-regulation of the expression of the cmyc gene and C-Myc protein	[39]
Aptamer	f-MWCNTs (–COOH)	MCF-7 (in vitro)	Internalization without affecting cell viability	[40]
Immunotherapy	f-SWCNTs (radiometal-ion chelates, E4G10)	Mice bearing LS174T (in vivo)	Reduction of tumor volume and improvement of median survival relative to controls	[41]
Immunotherapy	MWCNTs	Mice bearing H22 liver cancer (in vivo)	Promotion of inflammatory cytokine production and stimulation of macrophage phagocytosis	[42]
Immunotherapy	MWCNTs	Mice bearing GL261 (in vivo)	Increase of the macrophage influx into the glioma cells	[43]
Immunotherapy	f-SWCNTs (CpG)	Mice bearing glioma (in vivo)	Improvement of the survival of glioma bearing mice	[44]
Immunotherapy	f-bundled CNTs (MHC-1, αCD28, PLGA)	Mice bearing B16 melanoma (in vivo)	Action as an artificial antigen-presenting cell to expand T cells efficiently.	[45]
Protein	SWCNTs-biotin (Streptavidin)	HL60, human T cells (in vitro)	Consistent uptake pathway with endocytosis	[46]
Protein	f-MWCNTs (toxin protein RTA)	L-929, HeLa, HL7702, MCF-7, COS-7 (in vitro)	3-fold higher cell death rate than RTA alone	[47]
Fluorescence imaging	f-SWCNTs (sodium cholate and PEG)	Mouse (in vivo)	High-resolution microscopy imaging of tumor vessels below thick skin	[48]
Fluorescence imaging	f-SWCNTs (poly(maleic anhydride-alt-1-octadecene)-PEG)	Mice bearing 4T1 breast cancer (in vivo)	in vivo real-time fluorescence video imaging of mouse hind limb vasculatures	[49]
Fluorescence imaging	f-SWCNTs (M13 virus)	Mice bearing ovarian cancer (in vivo)	Identification of sub-millimeter tumors	[50]
Fluorescence imaging	f-SWCNTs (C_18_-PMH-mPEG)	Mice bearing 4T1 breast cancer (in vivo)	Imaging of tumors at an intravenous injection of ∼4 μg SWCNT material	[51]
Raman imaging	f-DWCNTs (folic acid)	T24 (in vitro)	Application of the location mapping of carcinoma cells and tracking of their movements	[52]
Raman imaging	f-SWCNTs (RGD)	Mice bearing U87MG tumor (in vivo)	Accumulation for >72 h in tumor area	[53]
Raman imaging	f-SWCNTs (RGD)	Mice bearing U87MG tumor (in vivo)	4-fold higher tumor binding than cyclo-(Arg-Ala-Asp-d-Phe-Lys)(RAD)-SWCNTs	[54]
Raman imaging	f-SWCNTs (Erbitux, RGD, anti-CEA, Rituxan, Herceptin)	MDA-MB-468, U87MG, LS174T, Raji, and BT474 (in vitro)	Feasibility of SWCNTs as NIR Raman tags for multi-color optical imaging and detection	[55]
Photoacoustic imaging	f-SWCNTs (Indocyanine Green dye-RGD)	Mice bearing U87MG tumor (in vivo)	sub-nanomolar sensitivities in living tissues and 20-fold higher cancer cell detection than previously-reported SWCNTs	[56]
Photoacoustic imaging	f-SWCNTs (gold-folic acid)	Mice bearing tumor (in vivo)	Improvement of detection sensitivity and specificity	[57]
Photoacoustic imaging	f-SWCNTs (Indocyanine Green dye)	-	2-fold higher signal amplitudes than SWCNTs	[58]
Photoacoustic imaging	f-SWCNTs (Albumin/Ce6-EB)	Mice bearing SCC-7 tumor (in vivo)	long blood circulating behavior (48 h)	[59]
Magnetic resonance imaging	f-MWCNTs (Gd chelate)	Mice (in vivo)	in vivo feasibility studies using MRI (300 MHz)	[60]
Magnetic resonance imaging	f-SWCNTs (Gd ion)	Mice (in vivo)	Successful imaging of the uptake of Gd-CNTs in the liver and spleen	[61]
Magnetic resonance imaging	f-MWCNTs (CoFe_2_O_4_)	HeLa (in vitro)	Favor as T_2_ contrast agent	[62]
Magnetic resonance imaging	f-MWCNTs (iron NP and Gd ion)	-	Dual function of MRI and magnetic hyperthermia cancer therapy	[63]

**Table 2:** Comparisons of material-based features between CNTs and polymer-based nanoparticles (NPs).

	Control of size	Cellular uptake	Drug loading	Functional surface	Water dispersibility	Toxicity
Carbon nanotubes	Moderate	Fast (needle-like shape)	High (high aspect ratio)	High (high aspect ratio and inner/outer surface)	Low (hydrophilic polymer coating or conjugation)	Moderate
Polymer-based NPs	Easy	Moderate	Low (encapsulation)	Moderate	Moderate (hydrophilic polymer)	Low

## References

[1] Ma D.D., Yang W.X. (2016). Engineered nanoparticles induce cell apoptosis: Potential for cancer therapy. Oncotarget.

[2] Luo J., Solimini N.L., Elledge S.J. (2009). Principles of cancer therapy: Oncogene and non-oncogene addiction. Cell.

[3] Hanahan D., Weinberg R.A. (2000). The hallmarks of cancer. Cell.

[4] Ariga K., Minami K., Ebara M., Nakanishi J. (2016). What are the emerging concepts and challenges in nano? Nanoarchitectonics, hand-operating nanotechnology and mechanobiology. Polym. J..

[5] Aono M., Ariga K. (2016). The way to nanoarchitectonics and the way of nanoarchitectonics. Adv. Mater..

[6] Ariga K., Ji Q.M., Nakanishi W., Hill J.P., Aono M. (2015). Nanoarchitectonics: A new materials horizon for nanotechnology. Mater. Horiz..

[7] Nakanishi W., Minami K., Shrestha L.K., Ji Q.M., Hill J.P., Ariga K. (2014). Bioactive nanocarbon assemblies: Nanoarchitectonics and applications. Nano Today.

[8] Ariga K., Kawakami K., Ebara M., Kotsuchibashi Y., Ji Q.M., Hill J.P. (2014). Bioinspired nanoarchitectonics as emerging drug delivery systems. New J. Chem..

[9] Moghimi S.M., Hunter A.C., Murray J.C. (2001). Long-circulating and target-specific nanoparticles: Theory to practice. Pharmacol. Rev..

[10] Zhang L., Gu F.X., Chan J.M., Wang A.Z., Langer R.S., Farokhzad O.C. (2008). Nanoparticles in medicine: Therapeutic applications and developments. Clin. Pharmacol. Ther..

[11] Iijima S. (1991). Helical microtubules of graphitic carbon. Nature.

[12] Lainioti G.C., Bounos G., Voyiatzis G.A., Kallitsis J.K. (2016). Enhanced water vapor transmission through porous membranes based on melt blending of polystyrene sulfonate with polyethylene copolymers and their cnt nanocomposites. Polymers.

[13] Elhissi A., Ahmed W., Dhanak V.R., Subramani K. (2012). Carbon nanotubes in cancer therapy and drug delivery. Micro Nano Technol..

[14] Wu W., Li R.T., Bian X.C., Zhu Z.S., Ding D., Li X.L., Jia Z.J., Jiang X.Q., Hu Y.Q. (2009). Covalently combining carbon nanotubes with anticancer agent: Preparation and antitumor activity. ACS Nano.

[15] Sahoo N.G., Bao H., Pan Y., Pal M., Kakran M., Cheng H.K., Li L., Tan L.P. (2011). Functionalized carbon nanomaterials as nanocarriers for loading and delivery of a poorly water-soluble anticancer drug: A comparative study. Chem. Commun..

[16] Tian Z., Yin M., Ma H., Zhu L., Shen H., Jia N. (2011). Supramolecular assembly and antitumor activity of multiwalled carbon nanotube-camptothecin complexes. J. Nanosci. Nanotechnol..

[17] Tripisciano C., Rummeli M.H., Chen X.C., Borowiak-Palen E. (2010). Multi-wall carbon nanotubes—A vehicle for targeted irinotecan drug delivery. Phys. Status Solidi B.

[18] Ali-Boucetta H., Al-Jamal K.T., McCarthy D., Prato M., Bianco A., Kostarelos K. (2008). Multiwalled carbon nanotube-doxorubicin supramolecular complexes for cancer therapeutics. Chem. Commun..

[19] Liu Z., Sun X.M., Nakayama-Ratchford N., Dai H.J. (2007). Supramolecular chemistry on water-soluble carbon nanotubes for drug loading and delivery. ACS Nano.

[20] Dinan N.M., Atyabi F., Rouini M.R., Amini M., Golabchifar A.A., Dinarvand R. (2014). Doxorubicin loaded folate-targeted carbon nanotubes: Preparation, cellular internalization, in vitro cytotoxicity and disposition kinetic study in the isolated perfused rat liver. Mater. Sci. Eng. C.

[21] Zhang X., Meng L., Lu Q., Fei Z., Dyson P.J. (2009). Targeted delivery and controlled release of doxorubicin to cancer cells using modified single wall carbon nanotubes. Biomaterials.

[22] Li R.B., Wu R.A., Zhao L.A., Hu Z.Y., Guo S.J., Pan X.L., Zou H.F. (2011). Folate and iron difunctionalized multiwall carbon nanotubes as dual-targeted drug nanocarrier to cancer cells. Carbon.

[23] Dhar S., Liu Z., Thomale J., Dai H.J., Lippard S.J. (2008). Targeted single-wall carbon nanotube-mediated pt(iv) prodrug delivery using folate as a homing device. J. Am. Chem. Soc..

[24] Hampel S., Kunze D., Haase D., Kramer K., Rauschenbach M., Ritschel M., Leonhardt A., Thomas J., Oswald S., Hoffmann V. (2008). Carbon nanotubes filled with a chemotherapeutic agent: A nanocarrier mediates inhibition of tumor cell growth. Nanomedicine.

[25] Liu Z., Chen K., Davis C., Sherlock S., Cao Q.Z., Chen X.Y., Dai H.J. (2008). Drug delivery with carbon nanotubes for in vivo cancer treatment. Cancer Res..

[26] Lay C.L., Liu H.Q., Tan H.R., Liu Y. (2010). Delivery of paclitaxel by physically loading onto poly(ethylene glycol) (peg)-graft-carbon nanotubes for potent cancer therapeutics. Nanotechnology.

[27] Singh R., Pantarotto D., McCarthy D., Chaloin O., Hoebeke J., Partidos C.D., Briand J.P., Prato M., Bianco A., Kostarelos K. (2005). Binding and condensation of plasmid DNA onto functionalized carbon nanotubes: Toward the construction of nanotube-based gene delivery vectors. J. Am. Chem. Soc..

[28] Geyik C., Evran S., Timur S., Telefoncu A. (2014). The covalent bioconjugate of multiwalled carbon nanotube and amino-modified linearized plasmid DNA for gene delivery. Biotechnol. Prog..

[29] Liu G.L., Wang Y., Hu Y., Yu X.B., Zhu B., Wang G.X. (2016). Functionalized multi-wall carbon nanotubes enhance transfection and expression efficiency of plasmid DNA in fish cells. Int. J. Mol. Sci..

[30] Karmakar A., Bratton S.M., Dervishi E., Ghosh A., Mahmood M., Xu Y., Saeed L.M., Mustafa T., Casciano D., Radominska-Pandya A. (2011). Ethylenediamine functionalized-single-walled nanotube (f-SWNT)-assisted in vitro delivery of the oncogene suppressor *p53* gene to breast cancer MCF-7 cells. Int. J. Nanomed..

[31] Pantarotto D., Singh R., McCarthy D., Erhardt M., Briand J.P., Prato M., Kostarelos K., Bianco A. (2004). Functionalized carbon nanotubes for plasmid DNA gene delivery. Angew. Chem. Int. Ed..

[32] Gao L.Z., Nie L., Wang T.H., Qin Y.J., Guo Z.X., Yang D.L., Yan X.Y. (2006). Carbon nanotube delivery of the *GFP* gene into mammalian cells. Chembiochem.

[33] Wang X., Ren J., Qu X. (2008). Targeted rna interference of cyclin a2 mediated by functionalized single-walled carbon nanotubes induces proliferation arrest and apoptosis in chronic myelogenous leukemia k562 cells. ChemMedChem.

[34] Podesta J.E., Al-Jamal K.T., Herrero M.A., Tian B., Ali-Boucetta H., Hegde V., Bianco A., Prato M., Kostarelos K. (2009). Antitumor activity and prolonged survival by carbon-nanotube-mediated therapeutic sirna silencing in a human lung xenograft model. Small.

[35] Zhang Z.H., Yang X.Y., Zhang Y., Zeng B., Wang Z.J., Zhu T.H., Roden R.B.S., Chen Y.S., Yang R.C. (2006). Delivery of telomerase reverse transcriptase small interfering RNA in complex with positively charged single-walled carbon nanotubes suppresses tumor growth. Clin. Cancer Res..

[36] Varkouhi A.K., Foillard S., Lammers T., Schiffelers R.M., Doris E., Hennink W.E., Storm G. (2011). SiRNA delivery with functionalized carbon nanotubes. Int. J. Pharm..

[37] Bartholomeusz G., Cherukuri P., Kingston J., Cognet L., Lemos R., Leeuw T.K., Gumbiner-Russo L., Weisman R.B., Powis G. (2009). In vivo therapeutic silencing of hypoxia-inducible factor 1 alpha (hif-1α) using single-walled carbon nanotubes noncovalently coated with siRNA. Nano Res..

[38] Guo C., Al-Jamal W.T., Toma F.M., Bianco A., Prato M., Al-Jamal K.T., Kostarelos K. (2015). Design of cationic multiwalled carbon nanotubes as efficient siRNA vectors for lung cancer xenograft eradication. Bioconjug. Chem..

[39] Pan B.F., Cui D.X., Xu P., Ozkan C., Feng G., Ozkan M., Huang T., Chu B.F., Li Q., He R. (2009). Synthesis and characterization of polyamidoamine dendrimer-coated multi-walled carbon nanotubes and their application in gene delivery systems. Nanotechnology.

[40] Van den Bossche J., Al-Jamal W.T., Tian B.W., Nunes A., Fabbro C., Bianco A., Prato M., Kostarelos K. (2010). Efficient receptor-independent intracellular translocation of aptamers mediated by conjugation to carbon nanotubes. Chem. Commun..

[41] Ruggiero A., Villa C.H., Holland J.P., Sprinkle S.R., May C., Lewis J.S., Scheinberg D.A., McDevitt M.R. (2010). Imaging and treating tumor vasculature with targeted radiolabeled carbon nanotubes. Int. J. Nanomed..

[42] Meng J., Yang M., Jia F.M., Kong H., Zhang W.Q., Wang C.Y., Xing J.M., Xie S.S., Xu H.Y. (2010). Subcutaneous injection of water-soluble multi-walled carbon nanotubes in tumor-bearing mice boosts the host immune activity. Nanotechnology.

[43] VanHandel M., Alizadeh D., Zhang L., Kateb B., Bronikowski M., Manohara H., Badie B. (2009). Selective uptake of multi-walled carbon nanotubes by tumor macrophages in a murine glioma model. J. Neuroimmunol..

[44] Ouyang M., White E.E., Ren H., Guo Q., Zhang I., Gao H., Yanyan S., Chen X., Weng Y., Da Fonseca A. (2016). Metronomic doses of temozolomide enhance the efficacy of carbon nanotube CPG immunotherapy in an invasive glioma model. PLoS ONE.

[45] Fadel T.R., Sharp F.A., Vudattu N., Ragheb R., Garyu J., Kim D., Hong E., Li N., Haller G.L., Pfefferle L.D. (2014). A carbon nanotube-polymer composite for T-cell therapy. Nat. Nanotechnol..

[46] Shi Kam N.W., Jessop T.C., Wender P.A., Dai H. (2004). Nanotube molecular transporters: Internalization of carbon nanotube-protein conjugates into mammalian cells. J. Am. Chem. Soc..

[47] Weng X.X., Wang M.Y., Ge J., Yu S.N., Liu B.H., Zhong J., Kong J.L. (2009). Carbon nanotubes as a protein toxin transporter for selective her2-positive breast cancer cell destruction. Mol. Biosyst..

[48] Welsher K., Liu Z., Sherlock S.P., Robinson J.T., Chen Z., Daranciang D., Dai H.J. (2009). A route to brightly fluorescent carbon nanotubes for near-infrared imaging in mice. Nat. Nanotechnol..

[49] Robinson J.T., Hong G.S., Liang Y.Y., Zhang B., Yaghi O.K., Dai H.J. (2012). In vivo fluorescence imaging in the second near-infrared window with long circulating carbon nanotubes capable of ultrahigh tumor uptake. J. Am. Chem. Soc..

[50] Ghosh D., Bagley A.F., Na Y.J., Birrer M.J., Bhatia S.N., Belcher A.M. (2014). Deep, noninvasive imaging and surgical guidance of submillimeter tumors using targeted m13-stabilized single-walled carbon nanotubes. Proc. Natl. Acad. Sci. USA.

[51] Antaris A.L., Robinson J.T., Yaghi O.K., Hong G.S., Diao S., Luong R., Dai H.J. (2013). Ultra-low doses of chirality sorted (6,5) carbon nanotubes for simultaneous tumor imaging and photothermal therapy. ACS Nano.

[52] Lamprecht C., Gierlinger N., Heister E., Unterauer B., Plochberger B., Brameshuber M., Hinterdorfer P., Hild S., Ebner A. (2012). Mapping the intracellular distribution of carbon nanotubes after targeted delivery to carcinoma cells using confocal Raman imaging as a label-free technique. J. Phys. Condens. Matter.

[53] Zavaleta C., de la Zerda A., Liu Z., Keren S., Cheng Z., Schipper M., Chen X., Dai H., Gambhir S.S. (2008). Noninvasive Raman spectroscopy in living mice for evaluation of tumor targeting with carbon nanotubes. Nano Lett..

[54] Smith B.R., Zavaleta C., Rosenberg J., Tong R., Ramunas J., Liu Z., Dai H.J., Gambhir S.S. (2013). High-resolution, serial intravital microscopic imaging of nanoparticle delivery and targeting in a small animal tumor model. Nano Today.

[55] Liu Z., Tabakman S., Sherlock S., Li X.L., Chen Z., Jiang K.L., Fan S.S., Dai H.J. (2010). Multiplexed five-color molecular imaging of cancer cells and tumor tissues with carbon nanotube raman tags in the near-infrared. Nano Res..

[56] De la Zerda A., Bodapati S., Teed R., May S.Y., Tabakman S.M., Liu Z., Khuri-Yakub B.T., Chen X.Y., Dai H.J., Gambhir S.S. (2012). Family of enhanced photoacoustic imaging agents for high-sensitivity and multiplexing studies in living mice. ACS Nano.

[57] Galanzha E.I., Shashkov E.V., Kelly T., Kim J.W., Yang L.L., Zharov V.P. (2009). In vivo magnetic enrichment and multiplex photoacoustic detection of circulating tumour cells. Nat. Nanotechnol..

[58] Nguyen V.P., Oh Y., Ha K., Oh J., Kang H.W. (2015). Enhancement of high-resolution photoacoustic imaging with indocyanine green-conjugated carbon nanotubes. Jpn. J. Appl. Phys..

[59] Xie L.S., Wang G.H., Zhou H., Zhang F., Guo Z.D., Liu C., Zhang X.Z., Zhu L. (2016). Functional long circulating single walled carbon nanotubes for fluorescent/photoacoustic imaging-guided enhanced phototherapy. Biomaterials.

[60] Richard C., Doan B.T., Beloeil J.C., Bessodes M., Toth E., Scherman D. (2008). Noncovalent functionalization of carbon nanotubes with amphiphilic GD3+ chelates: Toward powerful T1 and T2 MRI contrast agents. Nano Lett..

[61] Marangon I., Menard-Moyon C., Kolosnjaj-Tabi J., Beoutis M.L., Lartigue L., Alloyeau D., Pach E., Ballesteros B., Autret G., Ninjbadgar T. (2014). Covalent functionalization of multi-walled carbon nanotubes with a gadolinium chelate for efficient T-1-weighted magnetic resonance imaging. Adv. Funct. Mater..

[62] Wu H.X., Liu G., Wang X., Zhang J.M., Chen Y., Shi J.L., Yang H., Hu H., Yang S.P. (2011). Solvothermal synthesis of cobalt ferrite nanoparticles loaded on multiwalled carbon nanotubes for magnetic resonance imaging and drug delivery. Acta Biomater..

[63] Peci T., Dennis T.J.S., Baxendale M. (2015). Iron-filled multiwalled carbon nanotubes surface-functionalized with paramagnetic Gd (III): A candidate dual-functioning MRI contrast agent and magnetic hyperthermia structure. Carbon.

[64] Wong B.S., Yoong S.L., Jagusiak A., Panczyk T., Ho H.K., Ang W.H., Pastorin G. (2013). Carbon nanotubes for delivery of small molecule drugs. Adv. Drug Deliv. Rev..

[65] Yang F., Jin C., Yang D., Jiang Y.J., Li J., Di Y., Hu J.H., Wang C.C., Ni Q.X., Fu D.L. (2011). Magnetic functionalised carbon nanotubes as drug vehicles for cancer lymph node metastasis treatment. Eur. J. Cancer.

[66] Datir S.R., Das M., Singh R.P., Jain S. (2012). Hyaluronate tethered, “smart” multiwalled carbon nanotubes for tumor-targeted delivery of doxorubicin. Bioconjug. Chem..

[67] Shi X.Y., Wang S.H., Shen M.W., Antwerp M.E., Chen X.S., Li C., Petersen E.J., Huang Q.G., Weber W.J., Baker J.R. (2009). Multifunctional dendrimer-modified multiwalled carbon nanotubes: Synthesis, characterization, and in vitro cancer cell targeting and imaging. Biomacromolecules.

[68] Yu Y., Kong L.J., Li L., Li N.E., Yan P. (2015). Antitumor activity of doxorubicin-loaded carbon nanotubes incorporated poly(lactic-*co*-glycolic acid) electrospun composite nanofibers. Nanoscale Res. Lett..

[69] Tripisciano C., Kraemer K., Taylor A., Borowiak-Palen E. (2009). Single-wall carbon nanotubes based anticancer drug delivery system. Chem. Phys. Lett..

[70] Fonseca C., Simoes S., Gaspar R. (2002). Paclitaxel-loaded plga nanoparticles: Preparation, physicochemical characterization and in vitro anti-tumoral activity. J. Control. Release.

[71] Li C., Yu D.F., Inoue T., Yang D.J., Milas L., Hunter N.R., Kim E.E., Wallace S. (1996). Synthesis and evaluation of water-soluble polyethylene glycol-paclitaxel conjugate as a paclitaxel prodrug. Anti-Cancer Drug.

[72] Chan J.Y.W., Chu A.C.Y., Fung K.P. (2000). Inhibition of p-glycoprotein expression and reversal of drug resistance of human hepatoma HEPG2 cells by multidrug resistance gene (MDR1) antisense RNA. Life Sci..

[73] Suri S.S., Fenniri H., Singh B. (2007). Nanotechnology-based drug delivery systems. J. Occup. Med. Toxicol..

[74] Cheng J.P., Meziani M.J., Sun Y.P., Cheng S.H. (2011). Poly(ethylene glycol)-conjugated multi-walled carbon nanotubes as an efficient drug carrier for overcoming multidrug resistance. Toxicol. Appl. Pharm..

[75] Bates K., Kostarelos K. (2013). Carbon nanotubes as vectors for gene therapy: Past achievements, present challenges and future goals. Adv. Drug Deliv. Rev..

[76] Fortunati E., Bout A., Zanta M.A., Valerio D., Scarpa M. (1996). In vitro and in vivo gene transfer to pulmonary cells mediated by cationic liposomes. Biochim. Biophys. Acta.

[77] Rochat T., Morris M.A. (2002). Gene therapy for cystic fibrosis by means of aerosol. J. Aerosol. Med..

[78] Fischer D., Bieber T., Li Y., Elsasser H.P., Kissel T. (1999). A novel non-viral vector for DNA delivery based on low molecular weight, branched polyethylenimine: Effect of molecular weight on transfection efficiency and cytotoxicity. Pharm. Res..

[79] Coutelle C., Williamson R. (1996). Liposomes and viruses for gene therapy of cystic fibrosis. J. Aerosol. Med..

[80] Kostarelos K., Lacerda L., Pastorin G., Wu W., Wieckowski S., Luangsivilay J., Godefroy S., Pantarotto D., Briand J.P., Muller S. (2007). Cellular uptake of functionalized carbon nanotubes is independent of functional group and cell type. Nat. Nanotechnol..

[81] Kam N.W.S., Liu Z., Dai H.J. (2005). Functionalization of carbon nanotubes via cleavable disulfide bonds for efficient intracellular delivery of siRNA and potent gene silencing. J. Am. Chem. Soc..

[82] Dong H., Ding L., Yan F., Ji H., Ju H. (2011). The use of polyethylenimine-grafted graphene nanoribbon for cellular delivery of locked nucleic acid modified molecular beacon for recognition of microRNA. Biomaterials.

[83] Jia N.Q., Lian Q., Shen H.B., Wang C., Li X.Y., Yang Z.N. (2007). Intracellular delivery of quantum dots tagged antisense oligodeoxynucleotides by functionalized multiwalled carbon nanotubes. Nano Lett..

[84] Gooding M., Malhotra M., Evans J.C., Darcy R., O’Driscoll C.M. (2016). Oligonucleotide conjugates—Candidates for gene silencing therapeutics. Eur. J. Pharm. Biopharm..

[85] Gopinath S.C., Lakshmipriya T., Chen Y., Arshad M.K., Kerishnan J.P., Ruslinda A.R., Al-Douri Y., Voon C.H., Hashim U. (2016). Cell-targeting aptamers act as intracellular delivery vehicles. Appl. Microbiol. Biotechnol..

[86] McDevitt M.R., Chattopadhyay D., Kappel B.J., Jaggi J.S., Schiffman S.R., Antczak C., Njardarson J.T., Brentjens R., Scheinberg D.A. (2007). Tumor targeting with antibody-functionalized, radiolabeled carbon nanotubes. J. Nucl. Med..

[87] Yang L., Ng K.Y., Lillehei K.O. (2003). Cell-mediated immunotherapy: A new approach to the treatment of malignant glioma. Cancer Control.

[88] Parney I.F., Hao C., Petruk K.C. (2000). Glioma immunology and immunotherapy. Neurosurgery.

[89] Hussey S.L., Peterson B.R. (2002). Efficient delivery of streptavidin to mammalian cells: Clathrin-mediated endocytosis regulated by a synthetic ligand. J. Am. Chem. Soc..

[90] Welsher K., Sherlock S.P., Dai H.J. (2011). Deep-tissue anatomical imaging of mice using carbon nanotube fluorophores in the second near-infrared window. Proc. Natl. Acad. Sci. USA.

[91] Barone P.W., Baik S., Heller D.A., Strano M.S. (2005). Near-infrared optical sensors based on single-walled carbon nanotubes. Nat. Mater..

[92] Hong G.S., Lee J.C., Robinson J.T., Raaz U., Xie L.M., Huang N.F., Cooke J.P., Dai H.J. (2012). Multifunctional in vivo vascular imaging using near-infrared II fluorescence. Nat. Med..

[93] Liu Z.A., Li X.L., Tabakman S.M., Jiang K.L., Fan S.S., Dai H.J. (2008). Multiplexed multicolor Raman imaging of live cells with isotopically modified single walled carbon nanotubes. J. Am. Chem. Soc..

[94] Zavaleta C.L., Smith B.R., Walton I., Doering W., Davis G., Shojaei B., Natan M.J., Gambhir S.S. (2009). Multiplexed imaging of surface enhanced Raman scattering nanotags in living mice using noninvasive Raman spectroscopy. Proc. Natl. Acad. Sci. USA.

[95] Liu Z., Tabakman S., Welsher K., Dai H.J. (2009). Carbon nanotubes in biology and medicine: In vitro and in vivo detection, imaging and drug delivery. Nano Res..

[96] Hong G., Diao S., Antaris A.L., Dai H. (2015). Carbon nanomaterials for biological imaging and nanomedicinal therapy. Chem. Rev..

[97] Wang C., Ma X.X., Ye S.Q., Cheng L., Yang K., Guo L., Li C.H., Li Y.G., Liu Z. (2012). Protamine functionalized single-walled carbon nanotubes for stem cell labeling and in vivo raman/magnetic resonance/photoacoustic triple-modal imaging. Adv. Funct. Mater..

[98] He X.X., Gao J.H., Gambhir S.S., Cheng Z. (2010). Near-infrared fluorescent nanoprobes for cancer molecular imaging: Status and challenges. Trends Mol. Med..

[99] Chance B. (1998). Near-infrared images using continuous, phase-modulated, and pulsed light with quantitation of blood and blood oxygenation. Ann. N. Y. Acad. Sci..

[100] Ishizawa T., Fukushima N., Shibahara J., Masuda K., Tamura S., Aoki T., Hasegawa K., Beck Y., Fukayama M., Kokudo N. (2009). Real-time identification of liver cancers by using indocyanine green fluorescent imaging. Cancer.

[101] Sekijima M., Tojimbara T., Sato S., Nakamura M., Kawase T., Kai K., Urashima Y., Nakajima I., Fuchinoue S., Teraoka S. (2004). An intraoperative fluorescent imaging system in organ transplantation. Transp. Proc..

[102] Weissleder R., Tung C.H., Mahmood U., Bogdanov A. (1999). In vivo imaging of tumors with protease-activated near-infrared fluorescent probes. Nat. Biotechnol..

[103] Kim S., Lim Y.T., Soltesz E.G., De Grand A.M., Lee J., Nakayama A., Parker J.A., Mihaljevic T., Laurence R.G., Dor D.M. (2004). Near-infrared fluorescent type ii quantum dots for sentinel lymph node mapping. Nat. Biotechnol..

[104] Iizumi Y., Okazaki T., Ikehara Y., Ogura M., Fukata S., Yudasaka M. (2013). Immunoassay with single-walled carbon nanotubes as near-infrared fluorescent labels. ACS Appl. Mater. Interfaces.

[105] Lim Y.T., Kim S., Nakayama A., Stott N.E., Bawendi M.G., Frangioni J.V. (2003). Selection of quantum dot wavelengths for biomedical assays and imaging. Mol. Imaging.

[106] Diao S., Hong G.S., Robinson J.T., Jiao L.Y., Antaris A.L., Wu J.Z., Choi C.L., Dai H.J. (2012). Chirality enriched (12,1) and (11,3) single-walled carbon nanotubes for biological imaging. J. Am. Chem. Soc..

[107] Yi H.J., Ghosh D., Ham M.H., Qi J.F., Barone P.W., Strano M.S., Belcher A.M. (2012). M13 phage-functionalized single-walled carbon nanotubes as nanoprobes for second near-infrared window fluorescence imaging of targeted tumors. Nano Lett..

[108] Serpell C.J., Kostarelos K., Davis B.G. (2016). Can carbon nanotubes deliver on their promise in biology? Harnessing unique properties for unparalleled applications. ACS Cent. Sci..

[109] Jorio A., Pimenta M.A., Souza A.G., Saito R., Dresselhaus G., Dresselhaus M.S. (2003). Characterizing carbon nanotube samples with resonance raman scattering. New J. Phys..

[110] Hong G.S., Lee J.C., Jha A., Diao S., Nakayama K.H., Hou L.Q., Doyle T.C., Robinson J.T., Antaris A.L., Dai H.J. (2014). Near-infrared ii fluorescence for imaging hindlimb vessel regeneration with dynamic tissue perfusion measurement. Circ. Cardiovasc. Imaging.

[111] Hong G.S., Diao S., Chang J.L., Antaris A.L., Chen C.X., Zhang B., Zhao S., Atochin D.N., Huang P.L., Andreasson K.I. (2014). Through-skull fluorescence imaging of the brain in a new near-infrared window. Nat. Photonics.

[112] Diao S., Blackburn J.L., Hong G., Antaris A.L., Chang J., Wu J.Z., Zhang B., Cheng K., Kuo C.J., Dai H. (2015). Fluorescence imaging in vivo at wavelengths beyond 1500 nm. Angew. Chem. Int. Ed. Engl..

[113] Jorio A., Saito R., Hafner J.H., Lieber C.M., Hunter M., McClure T., Dresselhaus G., Dresselhaus M.S. (2001). Structural (n,m) determination of isolated single-wall carbon nanotubes by resonant Raman scattering. Phys. Rev. Lett..

[114] Rao A.M., Richter E., Bandow S., Chase B., Eklund P.C., Williams K.A., Fang S., Subbaswamy K.R., Menon M., Thess A. (1997). Diameter-selective raman scattering from vibrational modes in carbon nanotubes. Science.

[115] Heller D.A., Baik S., Eurell T.E., Strano M.S. (2005). Single-walled carbon nanotube spectroscopy in live cells: Towards long-term labels and optical sensors. Adv. Mater..

[116] Heller D.A., Jin H., Martinez B.M., Patel D., Miller B.M., Yeung T.K., Jena P.V., Hobartner C., Ha T., Silverman S.K. (2009). Multimodal optical sensing and analyte specificity using single-walled carbon nanotubes. Nat. Nanotechnol..

[117] Wang X.D., Pang Y.J., Ku G., Xie X.Y., Stoica G., Wang L.H.V. (2003). Noninvasive laser-induced photoacoustic tomography for structural and functional in vivo imaging of the brain. Nat. Biotechnol..

[118] Ku G., Wang L.H.V. (2005). Deeply penetrating photoacoustic tomography in biological tissues enhanced with an optical contrast agent. Opt. Lett..

[119] Hoelen C.G., de Mul F.F., Pongers R., Dekker A. (1998). Three-dimensional photoacoustic imaging of blood vessels in tissue. Opt. Lett..

[120] Yang K., Hu L.L., Ma X.X., Ye S.Q., Cheng L., Shi X.Z., Li C.H., Li Y.G., Liu Z. (2012). Multimodal imaging guided photothermal therapy using functionalized graphene nanosheets anchored with magnetic nanoparticles. Adv. Mater..

[121] Ku G., Zhou M., Song S.L., Huang Q., Hazle J., Li C. (2012). Copper sulfide nanoparticles as a new class of photoacoustic contrast agent for deep tissue imaging at 1064 nm. ACS Nano.

[122] Agarwal A., Huang S.W., O’Donnell M., Day K.C., Day M., Kotov N., Ashkenazi S. (2007). Targeted gold nanorod contrast agent for prostate cancer detection by photoacoustic imaging. J. Appl. Phys..

[123] De la Zerda A., Liu Z.A., Bodapati S., Teed R., Vaithilingam S., Khuri-Yakub B.T., Chen X.Y., Dai H.J., Gambhir S.S. (2010). Ultrahigh sensitivity carbon nanotube agents for photoacoustic molecular imaging in living mice. Nano Lett..

[124] De la Zerda A., Zavaleta C., Keren S., Vaithilingam S., Bodapati S., Liu Z., Levi J., Smith B.R., Ma T.J., Oralkan O. (2008). Carbon nanotubes as photoacoustic molecular imaging agents in living mice. Nat. Nanotechnol..

[125] Doan B., Meme S., Beloeil J., Merbach A., Helm L., Tóth É. (2013). General principles of MRI. The Chemistry of Contrast Agents in Medical Magnetic Resonance Imaging.

[126] Sitharaman B., Kissell K.R., Hartman K.B., Tran L.A., Baikalov A., Rusakova I., Sun Y., Khant H.A., Ludtke S.J., Chiu W. (2005). Superparamagnetic gadonanotubes are high-performance MRI contrast agents. Chem. Commun..

[127] Al Faraj A., Cieslar K., Lacroix G., Gaillard S., Canot-Soulas E., Cremillieux Y. (2009). In vivo imaging of carbon nanotube biodistribution using magnetic resonance imaging. Nano Lett..

[128] Al Faraj A., Fauvelle F., Luciani N., Lacroix G., Levy M., Cremillieux Y., Canet-Soulas E. (2011). In vivo biodistribution and biological impact of injected carbon nanotubes using magnetic resonance techniques. Int. J. Nanomed..

[129] Cerpa A., Kober M., Calle D., Negri V., Gavira J.M., Hernanz A., Briones F., Cerdan S., Ballesteros P. (2013). Single-walled carbon nanotubes as anisotropic relaxation probes for magnetic resonance imaging. Medchemcomm.

[130] Doan B.T., Seguin J., Breton M., Le Beherec R., Bessodes M., Rodriguez-Manzo J.A., Banhart F., Beloeil J.C., Scherman D., Richard C. (2012). Functionalized single-walled carbon nanotubes containing traces of iron as new negative MRI contrast agents for in vivo imaging. Contrast Media Mol. Imaging.

[131] Rivera E.J., Sethi R., Qu F.F., Krishnamurthy R., Muthupillai R., Alford M., Swanson M.A., Eaton S.S., Eaton G.R., Wilson L.J. (2012). Nitroxide radicals@us-tubes: New spin labels for biomedical applications. Adv. Funct. Mater..

[132] Sitharaman B., Jacobson B.D., Wadghiri Y.Z., Bryant H., Frank J. (2013). The magnetic, relaxometric, and optical properties of gadolinium-catalyzed single walled carbon nanotubes. J. Appl. Phys..

[133] Choi J.H., Nguyen F.T., Barone P.W., Heller D.A., Moll A.E., Patel D., Boppart S.A., Strano M.S. (2007). Multimodal biomedical imaging with asymmetric single-walled carbon nanotube/iron oxide nanoparticle complexes. Nano Lett..

[134] Dinesh B., Bianco A., Menard-Moyon C. (2016). Designing multimodal carbon nanotubes by covalent multi-functionalization. Nanoscale.

[135] Firme C.P., Bandaru P.R. (2010). Toxicity issues in the application of carbon nanotubes to biological systems. Nanomedicine.

[136] Bianco A., Kostarelos K., Prato M. (2011). Making carbon nanotubes biocompatible and biodegradable. Chem. Commun..

